# Arrhythmogenic cardiomyopathy diagnosis and management: a systematic review of clinical practice guidelines and recommendations with insights for future research

**DOI:** 10.1093/ehjqcco/qcaf029

**Published:** 2025-05-19

**Authors:** Lorena Iezzi, Anna Sorella, Kristian Galanti, Sabina Gallina, Anwar A Chahal, Barbara Bauce, Alberto Cipriani, Rui Providencia, Luis R Lopes, Fabrizio Ricci, Mohammed Y Khanji

**Affiliations:** Department of Neuroscience, Imaging and Clinical Sciences, G. D’Annunzio University of Chieti-Pescara, Chieti 66100, Italy; Department of Neuroscience, Imaging and Clinical Sciences, G. D’Annunzio University of Chieti-Pescara, Chieti 66100, Italy; Department of Neuroscience, Imaging and Clinical Sciences, G. D’Annunzio University of Chieti-Pescara, Chieti 66100, Italy; Department of Neuroscience, Imaging and Clinical Sciences, G. D’Annunzio University of Chieti-Pescara, Chieti 66100, Italy; University Cardiology Division, Heart Department, SS. Annunziata Polyclinic, Chieti 66100, Italy; Institute for Advanced Biomedical Technologies, G. D’Annunzio University of Chieti-Pescara, Via Luigi Polacchi, 11, Chieti 66100, Italy; Barts Heart Centre, St. Bartholomew’s Hospital, Barts Health NHS Trust, West Smithfield, London EC1A 7BE, UK; Center for Inherited Cardiovascular Diseases, Department of Cardiology, WellSpan Health, 30 Monument Rd, York, PA 17403, USA; William Harvey Research Institute, NIHR Barts Biomedical Centre, Queen Mary University of London, Charterhouse Square, London EC1M 6BQ, UK; Department of Cardiovascular Medicine, Mayo Clinic, 200 First Str, SW, Rochester, MN 55905, USA; Department of Cardiac, Thoracic and Vascular Sciences, University of Padua, Via Giustiniani, 2, Padua 35128, Italy; Department of Cardiac, Thoracic and Vascular Sciences, University of Padua, Via Giustiniani, 2, Padua 35128, Italy; Barts Heart Centre, St. Bartholomew’s Hospital, Barts Health NHS Trust, West Smithfield, London EC1A 7BE, UK; Barts Heart Centre, St. Bartholomew’s Hospital, Barts Health NHS Trust, West Smithfield, London EC1A 7BE, UK; Institute of Cardiovascular Science, University College London, London, UK; Department of Neuroscience, Imaging and Clinical Sciences, G. D’Annunzio University of Chieti-Pescara, Chieti 66100, Italy; University Cardiology Division, Heart Department, SS. Annunziata Polyclinic, Chieti 66100, Italy; Institute for Advanced Biomedical Technologies, G. D’Annunzio University of Chieti-Pescara, Via Luigi Polacchi, 11, Chieti 66100, Italy; Barts Heart Centre, St. Bartholomew’s Hospital, Barts Health NHS Trust, West Smithfield, London EC1A 7BE, UK; William Harvey Research Institute, NIHR Barts Biomedical Centre, Queen Mary University of London, Charterhouse Square, London EC1M 6BQ, UK; Department of Cardiology, Newham University Hospital, Barts Health NHS Trust, London EC1M 6BQ, UK

**Keywords:** Arrhythmogenic cardiomyopathy, Multimodality cardiovascular imaging, Risk stratification, Guidelines recommendations, Cardiogenetics

## Abstract

Arrhythmogenic cardiomyopathy (ACM) is addressed in international guidelines and expert consensus statements. To assist clinicians in their routine practice and support decision-making, we performed a systematic review of the most relevant guidelines and recommendations for ACM diagnosis and management. Our search, covering MEDLINE, EMBASE, and resources from scientific societies over the last 10 years, identified two guidelines and three consensus statements that met rigorous inclusion criteria for detailed analysis.

In the examined documents, key areas of agreement included the critical role of cardiac imaging for initial diagnosis and ongoing monitoring, genetic testing in index patients, ventricular arrhythmia management, catheter ablation indications, heart failure treatment strategies, and exercise recommendations. However, significant differences were found in definitions and diagnostic criteria for ACM, interpretation of phenocopies, management of family members, and criteria for implantable cardioverter defibrillator implantation. Additional discrepancies emerged regarding the role of multidisciplinary teams, non-cardiac surgical considerations, atrial fibrillation management, and reproductive issues.

Crucially, there remain considerable gaps in evidence, especially in areas such as the management and follow-up of patients with potential or borderline ACM diagnoses, as well as the care of their relatives. The clinical implications of genetic findings, along with the clinical management of left-dominant, biventricular phenotypes, and hot phases of disease, are also insufficiently addressed. Furthermore, a critical shortfall is the lack of externally validated risk assessment tools to guide clinical decision-making. Bridging these gaps could help guiding future research and guideline development towards improving patient outcomes.

## Graphical Abstract

**Graphical Abstract:**
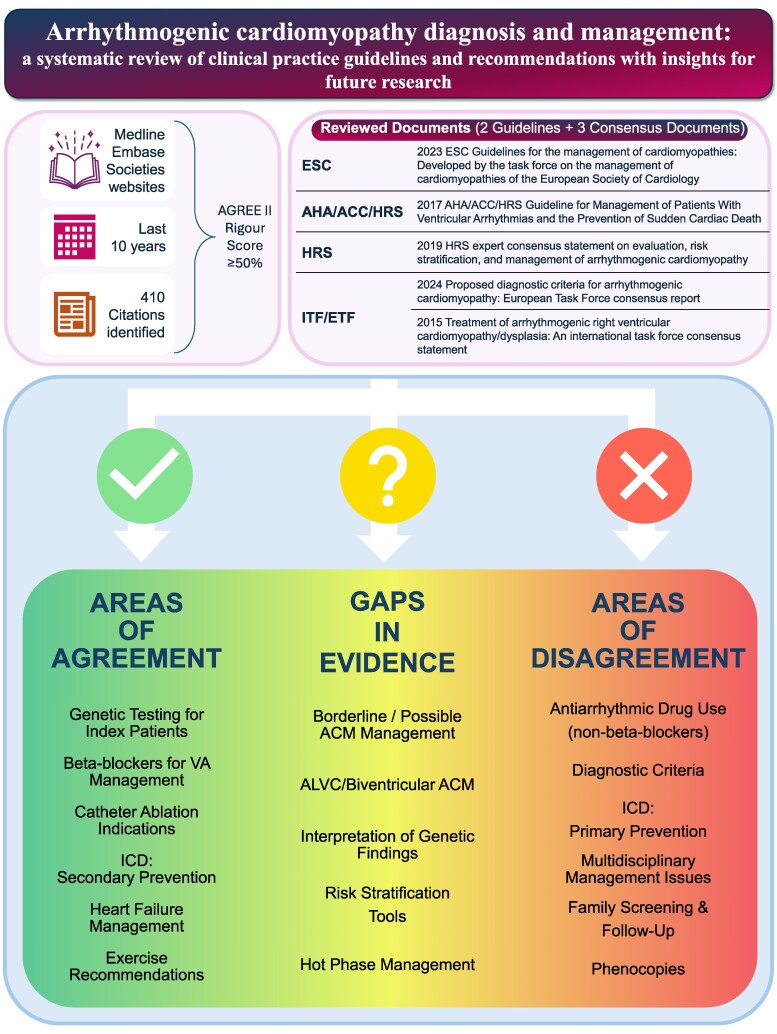

**Key Learning Points**

What is already known:

- The body of literature offering specific recommendations for the management of arrhythmogenic cardiomyopathy (ACM) remains relatively limited. Clinical guidelines and recommendations for ACM primarily address the establishment of diagnostic criteria, while specific recommendations for treatment, complication prevention, and family member management are less prominently featured.

What this study adds:

- This systematic review of clinical practice guidelines and recommendations on the diagnosis and management of ACM highlights areas of consensus, disagreement, and current gaps in the evidence. This document is intended to serve as a valuable resource for supporting clinicians in the management of ACM patients and to guide future research and guideline development efforts.

## Introduction

Arrhythmogenic cardiomyopathy (ACM) is a myocardial disease characterized by the replacement of healthy myocardium with fibrofatty tissue, leading to increased susceptibility to ventricular arrhythmias (VAs) and higher risk of sudden cardiac death (SCD), particularly among young individuals and athletes.^1^ Although ACM was initially believed to affect only the right ventricle,^2^ the discovery of associated genetic mutations and the involvement of the left ventricle led to a broader classification under the term ‘arrhythmogenic cardiomyopathy’.^3^ The condition frequently presents as a hereditary disorder, but its clinical manifestations vary significantly, even among individuals within the same family carrying identical pathogenic variants. Most of the implicated genes encode desmosomal proteins, including *PKP2*, *DSP*, *DSC2*, *DSG2*, and *JUP*, though some non-desmosomal genes have also been implicated.^4^ Mutations in desmosomal proteins lead to impaired adhesion between cardiac myocytes, making them more prone to detachment and cell death, and subsequent replacement by fibrofatty tissue. Specific genotype–phenotype correlations have been observed, indicating a predisposition to right, left, or biventricular involvement.^5^ The population frequency of ACM has been estimated at 1:1000 to 5000.^3^

The diagnostic criteria for arrhythmogenic right ventricular cardiomyopathy (ARVC) were initially established by the International Task Force (ITF) in 1994^6^ and later updated in 2010.^7^ In 2020, the ‘Padua criteria’ expanded the diagnostic framework to include left ventricular (LV) involvement.^8^ A key feature of these criteria is the use of cardiovascular magnetic resonance (CMR) imaging to detect myocardial scarring through late gadolinium enhancement (LGE). This technique is essential for identifying hallmark features of ACM, allowing for a more accurate diagnosis of the disease phenotype. Furthermore, it aids in distinguishing ACM from other non-scarring myocardial diseases, carrying significant prognostic and therapeutic implications.^3^ The Padua criteria were further refined in 2024 in the European Task Force consensus,^1^ reflecting the recognition that various phenocopies and genocopies can meet the diagnostic criteria and need to be accurately identified because their prognosis, treatment, and outcomes may be dependent on their underlying aetiology.

In clinical practice, the management of ACM focuses on the prevention of VAs and SCD using antiarrhythmic drugs, catheter ablation procedures, and implantable cardiac defibrillator (ICD) implantation.

Despite extensive research, the existing literature on ACM still appears to provide conflicting information and inconsistencies regarding its definition, diagnosis, and management. This issue is evident in both guidelines and consensus statements, where recommendations are predominantly derived from observational studies, follow-up registries, and expert opinions, resulting in a lower level of evidence, typically graded as Level B or C.^1,9–12^ Our goal was to perform a systematic review of rigorously developed guidelines and recommendations from major scientific societies. Beyond identifying areas of consensus and divergence, we sought to spotlight key knowledge gaps that demand further investigation. Addressing these gaps will be crucial to provide clarity for clinical management and shaping future research directions and guideline development.

## Methods

### Data sources and searches

We conducted a systematic review of English language clinical practice guidelines and recommendations for the management of ACM in adults. We searched MEDLINE and EMBASE on 21 May 2024 for guidelines published in the last 10 years. We also searched websites of organizations relevant to guideline development (Online Supplementary material online, *Table S1*). This systematic review was planned, conducted, and reported in agreement with the Preferred Reporting Items for Systematic Reviews and Meta-Analyses (PRISMA) recommendations.^13^

### Study selection

We included contemporary documents published by professional organizations, which made specific recommendations for ACM diagnosis and management in adults and met the Institute of Medicine’s definition of a guideline.^14^ If more than one guideline from the same organization existed, we considered the most recent one. Given the limited guidelines in this field, consensus statements were considered for this work. We developed a search syntax which served as a basis for the search strategy (Online Supplementary Material). Key search terms included: ‘arrhythmogenic cardiomyopathy’, ‘arrhythmogenic right ventricular cardiomyopathy’, ‘arrhythmogenic left ventricular cardiomyopathy’, ‘arrhythmogenic biventricular cardiomyopathy’, ‘recommendation’, and ‘guideline’.

### Data extraction and quality assessment

Titles and abstracts were assessed by two independent reviewers (L.I. and K.G.) using Rayyan.ai (https://www.rayyan.ai/). Articles were excluded if both reviewers agreed they were ineligible. Discrepancies were resolved by consensus after discussion. Both reviewers performed the final selection for full data extraction. We used the 23-item Appraisal of Guidelines for Research and Evaluation (AGREE) II instrument to determine the rigour of development for each guideline. The two reviewers independently rated the items, conforming to the instructions of the AGREE II tool. The average rigour scores were obtained by expressing the sum of the individual scores as a percentage of the maximum possible score. Reproducibility of the two reviewers’ scores was good, with an average intraclass correlation coefficient of 0.92 (see Supplementary material online, Online Supplementary material online, *Table S2*). Editorial independence from the funding body, external funding, and disclosure of relationships with industry by individual guideline group members were also assessed.

### Data synthesis and analysis

Two reviewers (L.I. and K.G.) extracted all relevant recommendations from the guidelines and consensus documents that had an AGREE II score equal to or greater than 50%. A recommendation matrix was produced.

## Results

**Figure 1:**
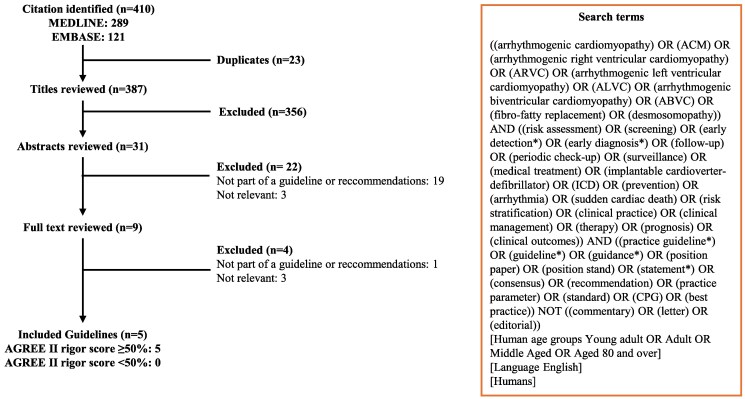
Summary of guideline search and review process. The number of guidelines at each step is indicated. AGREE II, Appraisal of Guidelines for Research and Evaluation II.

We retrieved 410 titles, of which 9 were potentially eligible. We retained two guidelines and three consensus statements on the management of ACM after review of the full manuscripts. All documents had a rigour score of ≥50% and form the object of this analysis (*Figure 1*). These were obtained from the following organization societies: European Society of Cardiology (ESC),^9^ European/International Task Force for diagnosis and treatment of Arrhythmogenic Cardiomyopathy (ETF/ITF),^1,10^ American Heart Association/American College of Cardiology/Heart Rhythm Society (AHA/ACC/HRS),^11^ and HRS.^12^  *Table 1* summarizes the selected guidelines and other recommendations along with rigour scores and conflicts of interest disclosed by the writing groups. The first table pertains to general recommendations for ACM diagnosis and treatment, while the second concerns specific diagnostic criteria for ACM. We decided to include both the ESC and the ETF/ITF among the European societies, as their respective guidelines and consensus statements provide different indications, primarily regarding diagnostic criteria and the definition of ACM. For the ETF/ITF, the key documents considered for ACM were the most recent consensus statement on diagnostic criteria, published in 2024 by Corrado *et al.*,^1^ and the 2015 consensus document on therapeutic recommendations for ACM.^10^ As for the HRS, this society is referenced in two documents: its own 2019 consensus statement^12^ and the earlier guideline published in 2017 by the AHA/ACC, which contains recommendations for the management of VAs.^11^ In this latter document, ACM is extensively discussed in a separate section, thus this guideline was considered the key document for the relevant American societies. No specific documents issued by Asian or Latin American societies were found in the literature, but it is noted in the HRS document that representatives from the Latin American Heart Rhythm Society, Asia Pacific Heart Rhythm Society, and Japanese Heart Rhythm Society contributed to its development.^12^

**Table 1:** Recommendations for arrhythmogenic cardiomyopathy diagnosis and management

Organization society	European Society of Cardiology (ESC)	American Heart Association/American College of Cardiology/Heart Rhythm Society (AHA/ACC/HRS)	Heart Rhythm Society (HRS)	European/International Task Force (ETF/ITF)
Document type	Clinical Practice Guidelines	Clinical Practice Guidelines	Consensus Statement	Consensus Statements
Region applied	Europe	USA	USA, Asia, Latin America	Europe
Year of publication	2023^9^	2017^11^	2019^12^	Diagnostic criteria: 2024^1^ Treatment: 2015^10^
AGREE II rigour score, %	93%	88%	78%	54%
Conflicts of interest	EI, SCI^a,b^	EI, SCI^a^	EI, SCI^a,b^	SCI^a^
Methods used to evaluate evidence	Systematic review	Systematic review	Systematic review	Systematic review
Methods used to formulate recommendations	Formal consensus	Formal consensus	Formal consensus	Formal consensus
Consideration of costs	Cost considered in strength of recommendation	Level of value provided when supporting data are available in the guideline, particularly for Class I and IIa recommendations. No level of value provided for specific ACM recommendations.	NR	NR
Definition of ACM	ARVC is defined as the presence of predominantly RV dilatation and/or dysfunction in the presence of histological involvement and/or ECG abnormalities in accordance with published criteria^6^. There is progressive myocardial atrophy with fibro-fatty replacement of the RV myocardium, but lesions can also be present in the LV myocardium.	Inherited cardiomyopathy that predominantly affects the right ventricle but can affect the left ventricle causing areas of myocardial replacement with fibrosis and adipose tissue that frequently causes VA and SCD	Arrhythmogenic heart muscle disorder not explained by ischaemic, hypertensive, or valvular heart disease	Heart muscle disease characterized by prominent non-ischaemic myocardial scarring predisposing to ventricular electrical instability, which may affect both ventricles, with some phenotypic variants being right-dominant, biventricular or left-dominant
Multidisciplinary team	Access to multidisciplinary teams with expertise in diagnosis and management for all patients and their relatives (**I-C**): Adult and paediatric cardiologists subspecialized in cardiogenetic conditions Cardiac imaging specialists (technicians, cardiologists, radiologists), including CMR experts Specialist nurses and/or genetic counsellors Clinical psychologists to support patients and their relatives Geneticists and bioinformaticians to interpret results of genetic investigations Expert pathologists to interpret findings by EMB and autopsy of individuals dying from a suspected inherited cardiac condition Transition of care from paediatric to adult services (**I-C**)	NR	NR	NR
Diagnostic criteria for ARVC	ARVC diagnosis should be suspected in adolescents or young adults with palpitations, syncope, or aborted sudden death; frequent VEs or VT of LBBB morphology; right pre-cordial TWI (V1–V3) in routine ECG testing; low QRS voltages in the peripheral leads and terminal activation delay in the right pre-cordial leads; right ventricular dilatation on 2D echocardiography. Revised Task Force Criteria for the diagnosis of ARVC (Marcus *et al.*^7^): *Definite*: 2 major OR 1 major + 2 minor OR 4 minor criteria from different categories *Borderline*: 1 major + 1 minor OR 3 minor criteria from different categories *Possible*: 1 major OR 2 minor criteria from different categories General endorsement of Padua criteria (Corrado *et al.*^8^), with acknowledgment of lack of external validation: No major or minor morpho-functional and/or structural LV criteria + *Definite*: 2 major OR 1 major + 2 minor OR 4 minor RV criteria from different categories (at least 1 major or minor morpho-functional and/or structural RV criteria) *Borderline*: 1 major + 2 minor OR 3 minor RV criteria from different categories (at least 1 major or minor morpho-functional and/or structural RV criteria) *Possible*: 1 major OR 2 minor RV criteria from different categories (at least 1 major or minor morpho-functional and/or structural RV criteria)	Presence of clinical symptoms along with the presence of Revised Task Force Criteria for the diagnosis of ARVC (Marcus *et al.*^7^): *Definite*: 2 major OR 1 major + 2 minor OR 4 minor criteria from different categories *Borderline*: 1 major + 1 minor OR 3 minor criteria from different categories *Possible*: 1 major OR 2 minor criteria from different categories	The diagnosis of ARVC should be considered in the following: patients with exercise-related palpitations and/or syncope; survivors of SCA (particularly during exercise); and individuals with frequent VEs (>500 in 24 h) and/or VT of LBBB morphology in the absence of other heart disease. Revised Task Force Criteria for the diagnosis of ARVC (Marcus *et al.*^7^): *Definite*: 2 major OR 1 major + 2 minor OR 4 minor criteria from different categories *Borderline*: 1 major + 1 minor OR 3 minor criteria from different categories *Possible*: 1 major OR 2 minor criteria from different categories	European Task Force Proposed Diagnostic Criteria for the diagnosis of ACM (Corrado *et al.* ^1^ ): No major or minor morpho-functional or structural (tissue-characterisation) LV criteria + *Definite*: 2 major OR 1 major + 2 minor OR 4 minor criteria for ARVC from different categories (at least 1 major or minor morpho-functional and/or structural RV criteria) *Borderline*: 1 major + 1 minor OR 3 minor criteria for ARVC from different categories (at least 1 major or minor morpho-functional and/or structural RV criteria) *Possible*: 1 major OR 2 minor criteria for ARVC from different categories (at least 1 major or minor morpho-functional and/or structural RV criteria)
Diagnostic criteria for ALVC	The NDLVC phenotype includes ALVC, left-dominant ARVC, or arrhythmogenic DCM. The term NDLVC is defined by the presence of non-ischaemic LV scarring or fatty replacement regardless of the presence of global or regional wall motion abnormalities, or isolated global LV hypokinesia without scarring. General endorsement of Padua criteria (Corrado *et al.*^8^), with acknowledgment of lack of external validation: No major or minor morpho-functional and/or structural RV criteria + ≥1 major structural LV criteria + Pathogenic or likely pathogenic ACM-causing gene mutation	NR	NR	European Task Force Proposed Diagnostic Criteria for the diagnosis of ACM (Corrado *et al.* ^1^ ): No major or minor morpho-functional or structural (tissue-characterisation) RV criteria + *Definite*: 2 major OR 1 major + 2 minor OR 4 minor criteria for ALVC from different categories (at least 1major or minor structural LV criteria) *Borderline*: 1 major + 1 minor OR 3 minor criteria for ALVC from different categories (at least 1 major or minor structural LV criteria) *Possible*: 1 major OR 2 minor criteria for ALVC from different categories (at least 1 major or minor structural LV criteria)
Diagnostic criteria for biventricular ACM	General endorsement of Padua criteria (Corrado *et al.* ^8^ ), with acknowledgment of lack of external validation: ≥1 major or minor morpho-functional and/or structural RV criteria + ≥1 major or minor morpho-functional or structural (tissue-characterization) LV criteria + *Definite*: 2 major OR 1 major + 2 minor OR 4 minor RV and LV criteria from different categories *Borderline*: 1 major + 2 minor OR 3 minor RV and LV criteria from different categories *Possible*: 1 major OR 2 minor RV and LV criteria from different categories	NR	NR	European Task Force Proposed Diagnostic Criteria for the diagnosis of ACM (Corrado *et al.* ^1^ ): ≥1 major or minor morpho-functional or structural (tissue-characterisation) RV criteria + ≥1 major or minor morpho-functional or structural (tissue-characterisation) LV criteria *Definite*: 2 major OR 1 major + 2 minor OR 4 minor criteria for ARVC and ALVC from different categories *Borderline*: 1 major + 1 minor OR 3 minor criteria for ARVC and ALVC from different categories *Possible*: 1 major OR 2 minor criteria for ARVC and ALVC from different categories
Diagnostic work-up	Systematic approach for suspected or established CMP: clinical evaluation, pedigree analysis, ECG, Holter monitoring, laboratory tests and multimodality imaging (**I-C**) Evaluation of family history and three-to-four-generation family tree (**I-C**)	Signal averaged ECG for diagnosis and risk stratification **(IIa-B NR)** Asymptomatic patients with clinical evidence of ARVC: EPS for risk stratification **(IIb-B NR)** Clinical screening with ECG, cardiac imaging, and ambulatory rhythm monitoring and/or exercise testing	Initial evaluation: clinical history, physical examination, detailed family history, 12-lead electrocardiogram (ECG), 2D echocardiography, ambulatory ECG monitoring, and CMR Additional testing: signal-averaged ECG, exercise ECG, pharmacological testing with isoproterenol, EBM, and EPS Family history covering at least 3 generations and clinical evaluation of relatives	EPS should be considered in the diagnosis and/or evaluation of patients with suspected ARVC (**IIa**) Programmed ventricular stimulation may be considered for arrhythmic risk stratification of asymptomatic ARVC patients (**IIb**) Endocardial voltage mapping may be considered in the diagnostic and prognostic evaluation of ARVC patients (**IIb**)
Assessment of symptoms	Palpitations Heart failure symptoms (possible many years after the onset of structural abnormalities)	NR	NR	NR
Laboratory tests	First-level tests for aetiological evaluation, disease severity assessment and extracardiac manifestations: calcium, CK, ferritin, full blood count, liver function, NT-proBNP, phosphate, proteinuria, renal function, serum iron, thyroid function, troponin, vitamin D (children) (**I-C**) Second-level tests in patients with extracardiac features for metabolic and syndromic causes detection: carnitine profile, free fatty acids, organ and non-organ specific serum autoantibodies, serum angiotensin-converting enzyme, thiamine, viral serology, urine organic acids and plasma amino acids (**IIa-C**) First level tests to be performed in every patient: C-reactive protein, liver function, NT-proBNP, renal function, troponin	NR	NR	NR
Multimodality imaging	Echocardiography: Comprehensive evaluation of cardiac dimensions, LV and RV systolic (global and regional) and LV diastolic function at initial evaluation and follow up (**I-B**) Myocardial deformation imaging (speckle tracking or tissue Doppler) with global longitudinal strain to detect subtle ventricular dysfunction Three-dimensional echocardiography to assess volumes of cardiac chambers Cardiac Magnetic Resonance: Contrast-enhanced CMR at initial evaluation (**I-B**), including cine imaging sequences, T2-weighted sequences, pre- and post-contrast T1 mapping, and LGE Contrast-enhanced CMR during follow up to monitor disease progression and aid risk stratification management (**IIa-C**): serial follow-up CMR, every 2–5 years depending on initial severity and clinical course Contrast-enhanced CMR in families in which a disease-causing variant has been identified for G+/P- family members (**IIa-C**) Contrast-enhanced CMR in P- family members without a genetic diagnosis (**IIb-C**) Computed Tomography and Nuclear Imaging: Contrast-enhanced cardiac CT for patients who have inadequate echocardiographic imaging and contraindications to CMR (**IIa-C**) CT-based imaging to exclude congenital or acquired CAD (**IIa-C**) 18F-FDG-PET scanning in suspected sarcoidosis (**IIa-C**): ARVC excluded if concomitant abnormal 18F-FDG-PET uptake in extracardiac tissues, or other clinical features suggestive of cardiac sarcoidosis CT-based imaging to rule out CAD, either as an alternative diagnosis or as a comorbidity affecting clinical manifestations and course	Cardiac MRI: It provides high-quality assessment of LV and RV function, size, regional wall motion abnormalities, and extent of scar and fibrosis (LGE) Suspected ARVC and VA or electrocardiographic abnormalities: cardiac MRI for establishing diagnosis and for risk stratification **(I-B NR)**	Echocardiography: Qualitative and quantitative assessment of ventricular function and cavity dimensions CMR: Accurate measurements of volumes, regional and global ventricular function Multidetector CT, RV angiography or radionuclide angiography: if CMR is contraindicated or not available Electrophysiology testing: refractory VA for ablation consideration and differentiation from RVOT tachycardia high-dose isoproterenol test: differentiate idiopathic VT or ventricular premature beats from ARVC	NR
Endomyocardial biopsy	When the results of other clinical investigations suggest myocardial inflammation, infiltration, or storage that cannot be identified by other mean (**IIa-C**) May be helpful to rule out myocarditis and sarcoidosis Consider electroanatomical voltage mapping-guided EMB in selected cases, particularly in case of negative CMR	NR	Useful in identifying systemic or inflammatory conditions that cause ACM (e.g. sarcoidosis, myocarditis) Limited due to false negatives secondary to patchy involvement and sampling error Electroanatomical voltage mapping guided EBM in areas of low voltage	NR
Phenocopies	Structural diseases: myocarditis, sarcoidosis, RV infarction, DCM, Chagas disease, pulmonary hypertension, and CHD with volume overload (such as Ebstein anomaly, atrial septal defect, and partial anomalous venous return, left-to-right shunt, and pericardial agenesis) Non-structural diseases: idiopathic RVOT VT, physiological adaptation to intensive training in competitive athletes	NR	Systemic disorders (e.g. sarcoidosis, amyloidosis), myocarditis, Chagas disease: considered as important cause of ACM ACM can overlap with DCM, HCM, RCM, LV non-compaction, non-sarcomeric HCM with arrhythmic presentation	ARVC Cardiac sarcoidosis (chronic granulomatous myocarditis) Chagas disease ALVC Post-acute or subacute/chronic viral myocarditis Cardiac sarcoidosis (chronic granulomatous myocarditis) Auto-immune multisystem diseases (systemic lupus erythematous; polymyositis/dermatomyositis; scleroderma) Chagas disease BIV Cardiac sarcoidosis (chronic granulomatous myocarditis) Chagas disease
Genetic counselling and testing	Genetic counselling: Counselling for families with an inherited or suspected inherited CMP, regardless of whether genetic testing is being considered (**I-B**) Testing with access to a multidisciplinary team: experts in genetic testing, sequence variant interpretation, clinical application of genetic testing (**I-B**) Pre- and post-test genetic counselling in all individuals undergoing genetic testing (**I-B**) Early in pregnancy if pre-natal diagnostic testing is to be pursued by the family (**I-C**) Reproductive genetic testing discussion for all families with a genetic diagnosis (**IIa-C**) Genetic testing for index patients: For CMP diagnosis, prognostication, therapeutic stratification, reproductive management or if cascade genetic testing is enabled (**I-B**) For a deceased individual at *post-mortem* if a genetic diagnosis would facilitate management of surviving relatives (**I-C**) In patients fulfilling diagnostic criteria when it will have a net benefit to the patient, considering the psychological impact and preference (**IIb-C**) Borderline phenotype not fulfilling diagnostic criteria after detailed assessment (**IIb-C**) Family members: Cascade genetic testing with pre- and post-test counselling in adult at-risk relatives if established confident genetic diagnosis in a family individual (**I-B**) Cascade genetic testing with pre- and post-test counselling in paediatric-at-risk relatives if established confident genetic diagnosis in a family individual (**IIa-B**) Testing for a familial variant of unknown significance in parents and/or affected relatives (**IIa-C**) Not recommended in a P- relative in the absence of a confident genetic diagnosis in the family (**III-C**)	Genetic counselling and genetic testing: Patients with clinically diagnosed or suspected ARVC, for diagnosis and for gene-specific targeted family screening **(IIa-B NR)** Family members: Selected first-degree relatives of patients with ARVC, if the proband has a disease-causing mutation: clinical screening along with genetic counselling and genetic testing (**I-B NR**)	Cardiac genetic test: interpretation by team of providers with expertise in genetics and cardiology **(IIa C-EO)** Comprehensive 3-generation family history **(I C-EO)** Reason for using results of genetic testing: Prenatal diagnostic or pre-implantation genetic diagnosis for pathogenic variants Genetic cascade screening in family members for pathogenic and likely- pathogenic variants Family members: Asymptomatic family members negative for the family’s pathogenic variant: dismissed from regular screening and educated to return if symptoms occur **(IIb C-EO)** Clinically unaffected family members should not be tested for a VUS found in the family	Repeat clinical assessment (every 2–3 years), mostly during adolescence and young adulthood, for healthy gene carriers and family members
Genetic assessment	Genes associated with monogenic, non-syndromic ARVC: Pathogenic/likely pathogenic variants: Very common (>10% of tested cases) with strong evidence: *PKP2* Common (1–10% of tested cases) with strong evidence: *DSC2, DSG2* Less common (<1% of tested cases) with strong evidence: *DSP, JUP, TMEM43* Less common (<1% of tested cases) with moderate evidence: *DES, PLN* *Pattern of inheritance*: autosomal dominant in the majority of ARVC families *Penetrance in genetic carriers*: age, gender, and physical activity dependent VUS: Less common (<1% of tested cases) with limited evidence: *ACTC1, CDH2, CTNNA3, LDB3, LMNA, MYBPC3, MYH7, MYL2, MYL3, RYR2, SCN5A, TGFB3, TJP1, TNNC1, TNNI3, TNNT2, TMP1, TTN* Less common (<1% of tested cases), evidence described, yet not classified/evaluated: *FNLC*	NR	Methods for screening genes: Sanger sequencing (single genes) Next-generation sequencing panel Whole exhome sequencing Whole genome sequencing Minimum set of genes: *BAG3* *DES* *DSC2* *DSG2* *DSP* *FLNC* *JUP* *LDB3* *LMNA* *NKX2-5* *PKP2* *PLN* *RBM20* *SCN5A* *TMEN43*	Desmosomal gene defects: *PKP2* (ARVC) *DSP* (ALVC-BIV-ARVC) * *DSC2* (ARVC-BIV) *DSG2* (ALVC-BIV) *JUP* (recessive ARVC-ALVC) * Non-desmosomal gene defects (genocopies) *TMEM43* (ARVC) *PLN* (ALVC-BIV-ARVC) *FLNC* (ALVC-BIV) *DES* (ALVC-BIV) *LMNA* (ALVC-BIV) *TGFB3* (ARVC) *CTNNA3* (ARVC) *CDH2* (ARVC) *SCN5A* (ARVC-ALVC) *DMD* (ALVC) ** *DMPK* (ALVC) ** *Cardio-cutaneous syndromes **Neuromuscular disorders
Surveillance plan	Multiparametric approach including ECG and echocardiography for all clinically stable patients every 1-to-2 year (**I-C**) Annual ambulatory ECG monitoring to aid in diagnosis, management, and risk stratification of ARVC **(I-C)** ECG and multimodality imaging in patients whenever there is a substantial or unexpected change in symptoms (**I-C**) Contrast-enhanced CMR during follow up to monitor disease progression and aid risk stratification management (**IIa-C**): serial follow-up CMR, every 2–5 years depending on initial severity and clinical course	NR	NR	Resting 12-lead ECG Echocardiography 24-h Holter monitoring exercise testing (for detection of effort-induced ventricular arrhythmias) To be performed on a regular basis (every 1–2 years) depending on the age, symptoms, and disease severity.
Management of ventricular arrhythmias	Antiarrhythmic treatment ARVC patients with VE, NSVT, and VT: beta-blockers **(I-C)** Failure of regular beta-blocker therapy to control arrhythmia-related symptoms: amiodarone **(IIa-C)** Failure of single agent treatment to control arrhythmia-related symptoms: flecainide in addition to beta-blockers **(IIa-C)** Identification and correction of any reversible cause and/or precipitating factor, such as electrolyte imbalances, ischaemia, hypoxaemia, or drugs Achievement of acute termination of sustained VA with electrical cardioversion, antiarrhythmic drugs, or pacing (initial choice depending on the haemodynamic tolerance, the underlying etiology, and the patient profile) Mild-to-moderate sedation to alleviate psychological distress and reduce sympathetic tone in electrical storm; consider deep/sedation/intubation in intractable electrical storm	Beta-blockers (Atenolol PO 25-100 mg qd or bid) ARVC and VA: **I-B NR** ARVC and no VA: **IIa-B NR** Ambulatory monitoring and/or exercise testing to assess adequacy of beta-blocking dose	Inappropriate ICD intervention resulting from sinus tachycardia, supraventricular tachycardia, or atrial fibrillation/flutter: beta-blockers **(I C-LD)** ACM who do not have an ICD: beta-blockers **(IIa C-EO)** Arrhythmic symptoms or to reduce ICD shocks: amiodarone **(IIb B-NR)**, sotalol **(IIb C-LD)** ACM, ICD, and preserved LV and RV function: flecainide in combination with beta-blockers and in absence of other antiarrhythmic drugs for control of VA refractory to other therapies **(IIb C-LD)**	Antiarrhythmic drugs: as an adjunct therapy to ICD in ARVC patients with frequent appropriate device discharges **(I)** to improve symptoms in patients with frequent premature ventricular beats and/or non-sustained VT **(IIa)** as an adjunct therapy to catheter ablation without a back-up ICD in selected ARVC patients with recurrent, haemodynamically stable VT **(IIb)** Not recommended in asymptomatic ARVC patients without documented ventricular arrhythmias and healthy gene carriers **(III)** Beta-blockers: ARVC patients with recurrent VT, appropriate ICD therapies, or inappropriate ICD interventions resulting from sinus tachycardia, supraventricular tachycardia, or atrial fibrillation/flutter with high-ventricular rate **(I)** should be considered in all patients with ARVC irrespective of arrhythmias **(IIa)** Prophylactic use of beta-blockers in healthy gene carriers is not recommended **(III)**
Catheter ablation	Incessant VT or frequent appropriate ICD interventions for VT despite pharmacological therapy with beta-blockers **(IIa-C),** with availability for epicardial approach guided by 3D electroanatomical mapping of VT Incessant VAs and electrical storm not responding to antiarrhythmic medication; in refractory cases or if VT ablation is either not indicated or not immediately available, may consider autonomic modulation (i.e. stellate ganglion block or cardiac sympathetic denervation) and/or MCS	Recurrent symptomatic sustained VT in whom a beta blocker is ineffective or not tolerated, catheter ablation with availability of a combined endocardial/epicardial approach **(IIa B-NR)**	Recurrent sustained monomorphic VT who has failed or are intolerant of amiodarone **(IIa B-NR)** with availability of combined endocardial/epicardial approach Recurrent symptomatic sustained VT in whom medical therapy has not failed **(IIb C-LD)** Symptomatic ventricular ectopy or NSVT in whom beta-blockers and/or antiarrhythmic medications are ineffective or not tolerated (**IIa C-LD**)	Incessant VT or frequent appropriate ICD interventions on VT despite maximal pharmacological therapy, including amiodarone **(I)** Epicardial approach to VT ablation in patients who fail one or more attempts of endocardial VT ablation **(I)** Incessant VT or frequent appropriate ICD interventions on VT who have failed pharmacological therapy other than amiodarone **(IIa)** Combined endocardial/epicardial VT ablation approach as an initial ablation strategy, provided that the operator and electrophysiologic laboratory are experienced performing epicardial VT ablation **(IIa)** Incessant VT or frequent appropriate ICD interventions on VT who have not failed pharmacological therapy and who do not wish to be treated with pharmacological therapy **(IIb)** First choice therapy without a back-up ICD for selected patients with drug-refractory, haemodynamically stable, single-morphology VT **(IIb)** Not recommended as an alternative to ICD for prevention of SCD **(III)**
ICD implantation	General recommendations: Only in patients who have an expectation of good quality survival > 1 year (**I-C**) Shared decision making that is evidence-based, considers a person’s individual preferences and ensures the person’s benefits and harms comprehension (**I-C**) Counselling on the risk of inappropriate shocks, implant complications and social, occupational and driving implications of the device (**I-C**) Not recommended in patients with incessant VAs, until VAs are controlled (**III-C**) Secondary prevention: In patients who have survived a cardiac arrest or who have recovered from a VA causing haemodynamic instability (**I-A** for ARVC, **I-C** for NDLVC including ALVC) In patients presenting with haemodynamically tolerated VT (**IIa-B**) Primary prevention: Consider high-risk features (arrhythmic syncope, NSVT, RVEF <40%, LVEF <45%, sustained monomorphic ventricular tachycardia at programmed electrical stimulation) to aid individualized decision-making for ICD implantation (**IIa-C**) Consider using the updated 2019 ARVC risk calculator to the shared decision-making (**IIa-B**) If a patient requires pacemaker implantation, consider comprehensive SCD risk stratification for eventual ICD implantation (**IIa-C**) Patients with DCM/NDLVC and LVEF ≤ 35% despite three months of optimized medical therapy (**IIa-A**) Patients with DCM/NDLVC, LVEF > 35% and high-risk genotypes (**IIa-C** in presence of additional risk factors, **IIb-C** in absence of additional risk factors**)** High risk genotypes: *LMNA, FLNC*-truncating variants, *TMEM43*: 5–10% annual SCD rate *PLN, DPS, RBM20*: 3–5% annual SCD rate Predictors of SCD: *LMNA*: estimated 5-year risk of life-threatening arrhythmias using *LMNA* risk score *FNLC*-truncating variants: LGE on CMR, LVEF < 45% *TMEM43*: male, female plus LVEF < 45% or NSVT or LGE on CMR or >200 VE on 24 h Holter ECG *PLN*: estimated 5-year risk of life-threatening arrhythmias using PLN risk score, LVEF < 45%, LGE on CMR, NSVT *DSP*: LVEF < 45%, LGE on CMR *RBM20*: LVEF < 45%, LGE on CMR Patients with NDLVC without genotype associated with high SCD risk and LVEF > 35% in presence of additional risk factors (e.g. syncope, LGE on CMR) **(IIb-C)**. Choice of ICD: Evaluate whether the patient could benefit from CRT (**I-A**) Subcutaneous defibrillators as an alternative to transvenous ones when pacing therapy for bradycardia, cardiac resynchronisation or antitachycardia pacing is not anticipated (**IIa-B**) Wearable ICD for adult patients with a secondary prevention indication who are temporarily not candidates for ICD implantation (**IIa-C**)	Patients with ARVC and an addition marker of increased risk of SCD (resuscitated SCA, sustained VT, significant ventricular dysfunction with RVEF or LVEF ≤35%), if meaningful survival greater than 1 year is expected **(I-B NR)** Patients with ARVC and syncope presumed due to VA, if meaningful survival greater than 1 year is expected **(IIa-B NR)**	Shared decision-making process between the patient and the physician, considering risks and benefits of the ICD over the potential longevity of the patient **(I C-EO)** Individuals with ACM and Cardiac arrest with VT or VF **(I B-NR)** SVT not haemodynamically tolerated **(I B-NR)** Syncope suspected to be due to VA **(IIa B-NR)** Haemodynamically tolerated SVT in ACM other than ARVC **(I B-NR)** in ARVC **(IIa B-NR)** *PLN* ACM and LVEF <45% or NSVT **(IIa B-NR)** *Lamin A/C* ACM and two or more of the following: **(IIa B-NR)** LVEF <45% Non-sustained VT Male sex *FLNC* ACM and LVEF < 45% **(IIa C-LD)** *Lamin A/C* ACM and an indication for pacing **(IIa C-LD)** ACM with LVEF≤35% and NYHA class II-III and an expected meaningful survival of greater than 1 year **(I B-R)** ACM with LVEF≤35% and NYHA class I and an expected meaningful survival of greater than 1 year **(IIa B-R)** ARVC and 3 major, 2 major and 2 minor, or 1 major and 4 minor risk factors for VA **(IIb B-NR):** Major criteria: non-sustained VT, inducibility to VT at EPS, LVEF≤49% Minor criteria: male sex, VEs/24 h > 1000, RV dysfunction, proband status, 2 or more desmosomal variants	High risk patients: estimated rate of life-threatening arrhythmic events ≥10%/year **(I)** Aborted SCD due to VF Sustained VT Severe dysfunction of RV (RV FAC ≤17% or RVEF ≤35%), LV (LVEF ≤35%), or both, irrespective of arrhythmias Intermediate risk patients: estimated rate of life-threatening arrhythmic events 1–10%/year ≥1 major risk factors **(IIa)** Syncope Non-sustained VT Haemodynamically stable, sustained VT Moderate dysfunction of RV (RV FAC 17–24% or RVEF 36–40%), LV (LVEF 36–45%), or both ≥1 minor risk factors **(IIb**, after a careful discussion of the long-term risks and benefits of ICD implantation) RV dilatation Right atrial dilatation Young age Male gender Complex genotype Inducible VT/VF Extent of electroanatomic scar on RV endocardial voltage mapping Fragmented electrograms on RV endocardial voltage mapping T-wave inversion in inferior leads Extent of T-wave inversion QRS fragmentation Precordial QRS amplitude ratio Low risk patients: estimated rate of life-threatening arrhythmic events <1%/year **(III)** No risk factors Healthy gene carriers Choice of ICD A single-chamber ICD system is recommended in order to minimize the incidence of long-term lead-related complications, mostly in young patients Leadless subcutaneous ICD: patient-specific indication, balancing lead-related complications with the likelihood of recurrent VT Additional cardiac resynchronisation for LVEF ≤35% and a wide QRS with a LBBB pattern
Heart failure management	HF medical therapy according to the 2021 ESC Guidelines for the diagnosis and treatment of acute and chronic heart failure Preventive HF medical therapy of asymptomatic carriers/early disease expression should be decided individually, as medication has not been proved to affect disease expression	NR	Left ventricular failure: as per 2013 (updated in 2016) AHA/ACC and ESC guidelines Right ventricular failure: Isosorbide dinitrate to reduce preload **(IIb C-EO)** ACEi or ARBs, beta-blockers, aldosterone antagonists, and diuretics **(IIa C-EO)** if symptoms due to RV dysfunction	For ARVC/D patients who developed right- and/or left-sided HF, standard pharmacological treatment with angiotensin-converting-enzyme inhibitors, angiotensin II receptor blockers, beta-blockers, and diuretics is recommended **(I)** Long-term oral anticoagulation is generally indicated for secondary prevention in patients with documented intracavitary thrombosis or venous/systemic thromboembolism **(I)** For ARVC/D patients with asymptomatic RV and/or LV dysfunction treatment with angiotensin-converting-enzyme inhibitors or angiotensin II receptor blockers may be considered **(IIb)** Prophylactic anticoagulation for primary prevention of thromboembolism on the basis of ventricular dilatation/dysfunction, either global or regional, is not recommended **(III)**
Orthotopic cardiac transplantation	Patients with advanced HF (NYHA Class III-IV) or intractable VA refractory to therapy, who do not have absolute contraindications (**I-C**)	NR	NR	Recommended as a final therapeutic option in ARVC patients with either severe, unresponsive congestive HF or recurrent episodes of VT/VF which are refractory to catheter (and surgical) ablation in experienced centres and/or ICD therapy
Left ventricular assist device therapy	Mechanical circulatory support therapy in patients with advanced HF (NYHA Class III-IV) despite optimal pharmacological and device treatment, who are otherwise suitable for heart transplantation (**IIa-B**) Mechanical circulatory support therapy in patients with advanced HF (NYHA Class III-IV) despite optimal pharmacological and device treatment, who are not eligible for heart transplantation or other surgical options, and without severe right ventricular dysfunction (**IIa-B**)	NR	NR	NR
Atrial fibrillation and atrial flutter management	Anticoagulation: According to cardio-embolic risk: **I B** if CHA_2_DS_2_-VASc score≥2 in men or ≥3 in women **IIa B** if CHA_2_DS_2_-VASc score = 1 in men or =2 in women Always if HF or LVEF ≤50% Long-term rate control: Beta-blockers (preferred) Verapamil or diltiazem (only if LVEF≥40%) AV node ablation + CRT or physiological pacing Long-term rhythm control: Rhythm control preferred in case of symptoms or/and HF or LV dysfunction Flecainide (associated with beta-blockers) Amiodarone, sotalol Ablation Symptom control: AF catheter ablation for rhythm control after one failed or intolerant class I or III antiarrhythmic drugs in patients with paroxysmal or persistent AF (**I-B**) AF catheter ablation to reverse LV dysfunction when tachycardia-induced component is highly probable (**I-B**) Maintenance of sinus rhythm rather than rate control at an early stage in AF patients without major risk factors for recurrence (**IIa-C**) AF catheter ablation as first-line rhythm control therapy in patients with paroxysmal or persistent AF as an alternative to class I or III antiarrhythmic drugs (**IIa-C**) AF catheter ablation in patients with AF, HF and/or LVEF ≤ 50% (**IIa-B**) Comorbidities and associated risk factors management: Modification of unhealthy lifestyle and targeted therapy of intercurrent conditions (**I-B**)	NR	AF, intracavitary thrombosis, or venous/systemic thromboembolism: anticoagulant therapy **(I B-NR)** LV or RV aneurysm: antithrombotic therapy **(IIb C-EO)**	NR
Relatives screening and follow up	Pathogenic/likely pathogenic variant in proband: Cascade clinical (with multiparametric approach including ECG and cardiac imaging) and genetic testing in first-degree relatives **(I)** Same P/LP variant as the proband +/− clinical phenotype: long- term follow-up **(I-B)** P/LP variant negative: discharge **(I-C)** Variant of unknown significance in proband: segregation analysis/functional studies where possible, consider indication for either P/LP variant or no variant/not performed genetic testing cases No variant/genetic testing not performed in proband: cascade clinical screening (initial evaluation with multiparametric approach including ECG and cardiac imaging) in first-degree relatives **(I-C)** Cardiomyopathy phenotype: long-term follow up **(I)** Normal phenotype: repeat screening at regular intervals with ECG and cardiac imaging **(IIa-C)** During cascade screening, clinical evaluation of close relatives when a first-degree relative has died (**IIa-C**)	NR	First-degree relatives: clinical evaluation every 1–3 years starting at the age of 10–12 years of age **(I B-NR)** Cardiovascular evaluation: 12-lead ECG, ambulatory ECG, and cardiac imaging **(I B-NR)** Exercise stress testing **(IIb C-LD)**	NR
Psychological support in patients and family members	Psychological support by appropriately trained health professionals to all individuals experiencing premature SCD of a family member (**I-B**) Psychological support by appropriately trained health professionals to all individuals who receive an ICD (**I-B**) Psychological support by appropriately trained health professionals to all individuals experiencing new diagnosis, exercise restrictions, symptomatic disease, genetic testing (**IIa-C**)	NR	NR	NR
Exercise recommendations	Regular low-to-moderate intensity exercise in all able individuals (**I-C**) Individualized risk assessment for exercise prescription (**I-C**) Avoidance of high-intensity exercise, including competitive sport, in genotype-positive/phenotype-negative individuals in families with ARVC **(IIb-C)** Moderate- and high-intensity exercise, including competitive sport, is not recommended in individuals with ARVC **(III-B)**	Patients with clinical diagnosis of ARVC: avoiding intense exercise is recommended **(I B-NR)**	Frequent high-intensity endurance exercise in individuals with ARVC **(III B-NR)** Genotype-positive phenotype-negative adolescents and adults: clinical counsel about association between competitive or frequent high-intensity endurance exercise and increased likelihood of developing ARVC and VA **(I B-NR)** Competitive exercise: regular competition and systematic intense training Moderate endurance exercise (class B): downhill skiing, figure skating, running (sprint), volleyball High endurance exercise (class C): long-distance running, cross-country skiing, rowing, basketball	It is recommended that patients with a definite diagnosis of ARVD not participate in competitive and/or endurance sports **(I)** Patients with a definite diagnosis of ARVC should be restricted from participation in athletic activities, with the possible exception of recreational low-intensity sports **(IIa)** Restriction from competitive sports activity may be considered in ARVC family members with a negative phenotype, either healthy gene carriers **(IIa)** or with unknown genotype **(IIb)**
Reproductive issues	Pre-pregnancy risk assessment and counselling in all women using the mWHO classification of maternal risk (**I-C**) Counselling on safe and effective contraception in all women of fertile age and their partners (**I-C**) Counselling on the risk of disease inheritance for all men and women before conception (**I-C**) Vaginal delivery in most women, unless there are obstetric indications for caesarean section, severe HF (LVEF < 30% or NYHA Class III-IV), or severe outflow tract obstructions, or women in labour on oral anticoagulants (**I-C**) Careful medication review for safety and tolerability in advance of pregnancy (**I-C**) Anticoagulation with LMWH or VKAs according to the stage of pregnancy in patients with AF (**I-C**) Beta-blockers continuation with close follow-up of fetal growth and conditions of the neonate, and if benefits outweigh risks (**IIa-C**) Genetic counselling and testing in patients with peripartum CMP (**IIa-C**)	NR	NR	NR
Non-cardiac surgery (NCS)	Peri-operative ECG monitoring for cardiomyopathies patients undergoing surgery (**I-C**) When scheduled for intermediate or high-risk NCS, re-evaluation of LV function with echocardiography and NT-proBNP/BNP levels assessment, unless recently performed (**I-B**) Performing ECG and transthoracic echocardiogram before NCS, regardless of symptoms, in all patients aged < 65 years with a first-degree relative with CMP (**I-C**)	NR	NR	NR
Management of cardiovascular risk factors	Identification and management of risk factors and concomitant diseases recommended as an integral part of the management (**I-C**)	NR	NR	NR

ACC, American College of Cardiology; ACEi, angiotensin-converting enzyme inhibitors; ACM, arrhythmogenic cardiomyopathy; AF, atrial fibrillation; AHA, American Heart Association; ALVC, arrhythmogenic left ventricular cardiomyopathy; ARBs, angiotensin2 receptor blockers; ARVC, arrhythmogenic right ventricular cardiomyopathy; BSA, body surface area; CAD, cardiac artery disease; CE-CMR, contrast-enhancement cardiac magnetic resonance; CK, creatin kinase; CMP, cardiomyopathy; CMR, cardiac magnetic resonance; CRT, cardiac resynchronization therapy; CT, computed tomography; DCM, dilated cardiomyopathy; EBM, endomyocardial biopsy; ECG, electrocardiogram; EDV, end-diastolic volume; EI, editorial independence declared; EPS, electrophysiology study; ESC, European Society of Cardiology; ETF/ITF, European/International Task Force; FAC, fractional area change; G, genotype; HCM, hypertrophic cardiomyopathy; HF, heart failure; HRS, Heart Rhythm Society; ICD, implantable cardiac defibrillator; LAS, low amplitude signal; LBBB, left bundle branch block; LGE, late gadolinium enhancement; LV, left ventricle; LVEDV, left ventricular end-diastolic volume; LVEF, left ventricle ejection fraction; MCS, mechanical cardiac support; MRI, magnetic resonance imaging; mWHO, modified World Health Organization; NCS, non-cardiac surgery; NR, not reported; NSVT, non-sustained ventricular tachycardia; P, phenotype; P/LP, pathogenic/likely-pathogenic; PLAX, parasternal long axis; PSAX, parasternal short axis; RBBB, right bundle branch block; RCM, restrictive cardiomyopathy; RV, right ventricle; RVEDV, right ventricular end-diastolic volume; RVEF, right ventricle ejection fraction; RVOT, right ventricular outflow tract; SCA, sudden cardiac arrest; SCD, sudden cardiac death; SCI^a^, statement about conflicts of interest of group members present, relationship with industry is reported by any group member; SCI^b^, statement about conflicts of interest of group members present, a group member is reported recused when a relevant area is under discussion; TWI, T wave inversion; VA, ventricular arrhythmia; VE, ventricular extrasystole; VF, ventricular fibrillation; VT, ventricular tachycardia.

### Areas of agreement

#### Role of cardiac imaging

All the included documents unanimously acknowledge the essential role of echocardiography and CMR in the diagnosis, initial assessment, and follow-up of patients with ACM. While minor variations exist, all documents consistently classify right ventricular morpho-functional abnormalities, such as the detection of akinesia, dyskinesia, or aneurysm, as well as global right ventricular dilation or systolic dysfunction, via echocardiography and/or CMR as a diagnostic criterion.^1,9,11,12^ Furthermore, CMR is pivotal in identifying myocardial fibrosis through LGE, which is considered a key structural criterion.^1,8^ In addition to establishing the diagnosis, echocardiography and CMR are consistently recommended for follow-up and risk stratification in ACM patients.^1,9,11,12^

#### Genetic counselling/testing for index patients

Genetic analysis is endorsed in all reviewed documents, with a Class I^1,9^ or IIa^11,12^ recommendation for diagnosing ACM in index patients. In parallel, genetic counselling is equally recommended, both before and after testing, with the same level of indication and consensus. This process necessitates a multidisciplinary team that includes geneticists and cardiologists, highly experienced in sequence variant interpretation and the clinical application of genetic results.^9,12^ Furthermore, the ESC guidelines also advocate for *post-mortem* genetic analysis when a genetic diagnosis could facilitate management of surviving family members.^9^

#### Management of ventricular arrhythmias: beta-blockers

Ventricular arrhythmias are a major complication of ACM. Antiarrhythmic therapy plays a key role in managing and preventing these arrhythmias, and all reviewed documents agree that beta-blockers are the preferred class of antiarrhythmics for both sustained and non-sustained VAs, regardless of haemodynamic tolerance.^9–12^ Beta-blockers are also recommended for the management of supraventricular arrhythmias when they are the cause of inappropriate ICD interventions and are strongly recommended in addition to an ICD in cases of frequent appropriate device shocks (Class I).^9–12^ The AHA/ACC guideline uniquely specifies the type and dosage of beta-blocker for ARVC, recommending atenolol at 25–100 mg once or twice daily.^11^ By contrast, sotalol is recommended as a Class IIb therapy exclusively by the HRS,^12^ which advocates its use in younger patients to avoid the long-term administration of amiodarone and mitigate its extracardiac adverse effects. However, this recommendation is supported by a low level of evidence(C), reflecting a lack of robust data in the literature to support this approach. In contrast, the ESC^9^ and AHA^11^ only acknowledge sotalol, underscoring the scarcity and inconsistency of the evidence regarding its use. Beta-blockers are also advised in ARVC, regardless of the presence of VAs or an ICD, though with a lower class of recommendation (Class IIa).^10–12^

#### Indications for catheter ablation

All societies agree on a Class IIa indication for catheter ablation in cases of incessant ventricular tachycardia (VT) or frequent appropriate ICD interventions despite antiarrhythmic therapy with beta-blockers, or in cases of intolerance to beta-blockers.^9–12^ Additionally, the HRS^12^ extends this indication to cases with a high burden of ventricular ectopy or non-sustained VT. The ETF/ITF and HRS further recommend catheter ablation for incessant VT in cases of amiodarone failure, with Class I and IIa indications, respectively.^10,12^ Moreover, all societies support catheter ablation using a combined endo-epicardial approach.^9–12^ Catheter ablation of VT is also recommended by the ETF/ITF and HRS with a Class IIb indication when antiarrhythmic therapy has not failed or is not desired.^10,12^

#### ICD implantation for secondary prevention

All guidelines agree on the importance of shared decision-making between clinicians and patients.^9–12^ There is unanimous consensus on the indications for ICD implantation in the context of secondary prevention. Specifically, ICDs are strongly recommended (Class I) for patients who have survived SCD or experienced VAs leading to haemodynamic instability. Additionally, a Class IIa indication is recognized for individuals who have recovered from haemodynamically stable VT.^9–12^ Notably, the HRS guidelines elevate the recommendation to a Class I status for patients with arrhythmogenic left ventricular cardiomyopathy (ALVC) or biventricular arrhythmogenic cardiomyopathy (BivACM), when presenting with left ventricular ejection fraction (LVEF) 35% or lower and NYHA class II-III symptoms.^12^

#### Heart failure management

The reviewed documents unanimously recommend the use of angiotensin-converting enzyme inhibitors, angiotensin II receptor blockers, beta-blockers, SGLT2 inhibitors, and diuretics for treating heart failure, consistent with the heart failure guidelines issued by ESC in 2021^15^ and by AHA/ACC in 2016.^16^ Additionally, HRS document specify a Class IIb indication for isosorbide dinitrate to reduce preload in cases of right ventricular failure.^12^

#### Exercise recommendations

There is consensus in the guidelines and recommendations reviewed regarding the contraindication of high-intensity physical activity and endurance sports in patients with a definite diagnosis of ARVC.^9–12^ The ESC assigns a Class I indication for regular low-to-moderate intensity exercise in all capable individuals.^9^ In contrast, the ETF/ITF considers recreational low-intensity sports a possible exception to exercise restrictions (Class IIa),^10^ while the HRS and AHA/ACC do not address this issue.

### Areas of disagreement

#### Definition of ACM

While all documents agree on defining ACM as a heart muscle disorder characterized by non-ischaemic histological abnormalities, particularly the replacement of myocardium with fibrofatty tissue, which predisposes to ventricular electrical instability, VAs, and SCD, notable variations exist in how they emphasize the different phenotypes of ACM. These differences are particularly pronounced in the localization of pathological alterations (whether right, left, or biventricular).

The ESC guidelines, which take a phenotypic approach to various cardiomyopathies, primarily focus on ACM with predominant right ventricular involvement, categorizing left-dominant and biventricular forms to the section on non-dilated left ventricular cardiomyopathies (NDLVC).^9^ Similarly, the AHA/ACC and HRS documents define ACM as primarily involving the right ventricle, though they acknowledge potential LV involvement.^11^ In contrast, the ETF/ITF document describes ACM as a non-ischaemic myocardial scarring disorder that can affect both ventricles, giving equal importance to right-dominant (ARVC), left-dominant (ALVC), and biventricular (BivACM) phenotypic variants.^1^

#### Diagnostic criteria

The clinical diagnosis of ACM is particularly challenging due to the absence of a highly sensitive and specific diagnostic test. Instead, it relies on identifying a combination of multiple diagnostic criteria related to ventricular morpho-functional abnormalities, structural changes, ECG anomalies, arrhythmias, and family/genetic history. There is consensus among the ESC, AHA/ACC, and HRS in utilizing the diagnostic criteria proposed by Marcus *et al.*^9–12^ The ESC also supports the Padua Criteria,^8^ while acknowledging the lack of external validation.^9^ In contrast, the ETF/ITF proposed a refinement of the 2020 Padua criteria to improve the diagnosis of ACM with upgraded and internationally recognized criteria (*Tables 2* and *3*).

**Table 2:** Diagnostic criteria for arrhythmogenic cardiomyopathy

Organization Society	European Society of Cardiology (ESC)		American Heart Association/American College of Cardiology/Heart Rhythm Society (AHA/ACC/HRS)	Heart Rhythm Society (HRS)	European/International Task Force (ETF/ITF)
Diagnostic criteria for ARVC	ARVC diagnosis should be suspected in adolescents or young adults with palpitations, syncope, or aborted sudden death; frequent VEs or VT of LBBB morphology; right pre-cordial TWI (V1–V3) in routine ECG testing; low QRS voltages in the peripheral leads and terminal activation delay in the right pre-cordial leads; right ventricular dilatation on 2D echocardiography. Revised Task Force Criteria for the diagnosis of ARVC (Marcus *et al.*^7^): *Definite*: 2 major OR 1 major + 2 minor OR 4 minor criteria from different categories *Borderline*: 1 major + 1 minor OR 3 minor criteria from different categories *Possible*: 1 major OR 2 minor criteria from different categories General endorsement of Padua criteria (Corrado *et al.*^8^), with acknowledgment of lack of external validation: No major or minor morpho-functional or structural (tissue-characterisation) LV criteria + *Definite*: 2 major OR 1 major + 2 minor OR 4 minor RV criteria from different categories (at least 1 major or minor morpho-functional or structural RV criteria) *Borderline*: 1 major + 1 minor OR 3 minor RV criteria from different categories (at least 1 major or minor morpho-functional or structural RV criteria) *Possible*: 1 major OR 2 minor RV criteria from different categories (at least 1 major or minor morpho-functional or structural RV criteria)		Presence of clinical symptoms along with the presence of Revised Task Force Criteria for the diagnosis of ARVC (Marcus *et al.*^7^): *Definite*: 2 major OR 1 major + 2 minor OR 4 minor criteria from different categories *Borderline*: 1 major + 1 minor OR 3 minor criteria from different categories *Possible*: 1 major OR 2 minor criteria from different categories	The diagnosis of ARVC should be considered in the following: patients with exercise-related palpitations and/or syncope; survivors of SCA (particularly during exercise); and individuals with frequent VEs (>500 in 24 h) and/or VT of LBBB morphology in the absence of other heart disease. Revised Task Force Criteria for the diagnosis of ARVC (Marcus *et al.*^7^): *Definite*: 2 major OR 1 major + 2 minor OR 4 minor criteria from different categories *Borderline*: 1 major + 1 minor OR 3 minor criteria from different categories *Possible*: 1 major OR 2 minor criteria from different categories	European Task Force Proposed Diagnostic Criteria for the diagnosis of ACM (Corrado *et al.* ^1^ ): No major or minor morpho-functional or structural (tissue-characterisation) LV criteria + *Definite*: 2 major OR 1 major + 2 minor OR 4 minor criteria for ARVC (at least 1 major or minor morpho-functional or structural RV criteria) *Borderline*: 1 major + 1 minor OR 3 minor criteria for ARVC (at least 1 major or minor morpho-functional or structural RV criteria) *Possible*: 1 major OR 2 minor criteria for ARVC (at least 1 major or minor morpho-functional or structural RV criteria)
Morpho-functional ventricular abnormalities	*Revised Task Force Criteria for the diagnosis of ARVC (Marcus* et al.^7^*):* *Major (by 2D echo)* Regional RV akinesia, dyskinesia, or aneurysm AND one of the following (end diastole): PLAX view RVOT ≥32 mm PLAX view RVOT corrected for body size (PLAX/BSA) ≥19 mm/m^2^ PSAX view RVOT ≥36 mm PSAX view RVOT corrected for body size (PLAX/BSA) ≥21 mm/m^2^ OR FAC ≤33% *Major (by MRI)* Regional RV akinesia, dyskinesia, or dyssynchronous RV contraction AND one of the following: RVEDV/BSA ≥110 mL/m^2^ (male) or ≥100 mL/m^2^ (female) OR RVEF ≤40% *Major (by RV angiography)* Regional RV akinesia, dyskinesia, or dyssynchronous RV contraction *Minor (by 2D echo)* Regional RV akinesia OR dyskinesia AND one of the following (end diastole): PLAX view RVOT ≥29–≤32 mm PLAX view RVOT corrected for body size (PLAX/BSA) ≥16–≤19 mm/m^2^ PSAX view RVOT ≥32–≤36 mm PSAX view RVOT corrected for body size (PLAX/BSA) ≥18–≤21 mm/m^2^ OR FAC >33%–≤40% *Minor (by MRI)* Regional RV akinesia OR dyskinesia OR dyssynchronous RV contraction AND one of the following: RVEDV/BSA ≥100–<110 mL/m^2^ (male) or ≥90–<100 mL/m^2^ (female) OR RVEF >40%–≤45%	*Padua criteria* (Corrado *et al.*^8^): *Major (by 2D echo, CMR or angiography)* Regional RV akinesia, dyskinesia, or bulging plus one of the following: global RV dilatation (increase of RVEDV according to the imaging test specific nomograms for age, sex and BSA) OR global RV systolic dysfunction (reduction of RVEF according to the imaging test specific nomograms for age and sex) *Minor (by 2D echo, CMR or angiography)* Regional RV akinesia, dyskinesia or aneurysm of RV free wall	*Major (by 2D echo)* Regional RV akinesia, dyskinesia, or aneurysm AND one of the following (end diastole): PLAX RVOT ≥32 mm (PLAX/BSA ≥19 mm/m^2^) PSAX RVOT ≥36 mm (PSAX/BSA ≥21 mm/m^2^) OR FAC ≤33% *Major (by MRI)* Regional RV akinesia, dyskinesia, or dyssynchronous RV contraction AND one of the following: RVEDV/BSA ≥110 mL/m^2^ (male), ≥100 mL/m^2^ (female) OR RVEF ≤40% *Major (by RV angiography)* Regional RV akinesia, dyskinesia, or dyssynchronous RV contraction *Minor (by 2D echo)* Regional RV akinesia OR dyskinesia AND one of the following (end diastole): PLAX RVOT ≥29–≤32 mm (PLAX/BSA ≥16–≤19 mm/m^2^) PSAX RVOT ≥32–≤36 mm (PSAX/BSA ≥18–≤21 mm/m^2^) OR FAC >33%–≤40% *Minor (by MRI)* Regional RV akinesia OR dyskinesia OR dyssynchronous RV contraction AND one of the following: RVEDV/BSA ≥100–<110 mL/m^2^ (male), ≥90–<100 mL/m^2^ (female) OR RVEF >40%–≤45%	*Major (by 2D echo, CMR or angiography)* Regional RV akinesia, dyskinesia, or aneurysm plus one of the following: global RV dilatation (increase of RVEDV according to the imaging test specific nomograms for age, sex and BSA, i.e. CMR: EDV/BSA >112 mL/m^2^ for women or >121 mL/m^2^ for men or >130 mL/m^2^ for athletes) OR global RV systolic dysfunction (reduction of RVEF according to the imaging test specific nomograms for age and sex, i.e. CMR: RVEF <51% for women or <52% for men and athletes) *Minor (by 2D echo, CMR or angiography)* Regional RV akinesia, dyskinesia or aneurysm of RV free wall	*Major (by 2D echo)* Regional RV akinesia, dyskinesia, or aneurysm AND one of the following (end diastole): PLAX RVOT ≥32 mm (PLAX/BSA ≥19 mm/m^2^) PSAX RVOT ≥36 mm (PSAX/BSA ≥21 mm/m^2^) OR FAC ≤33% *Major (by MRI)* Regional RV akinesia, dyskinesia, or dyssynchronous RV contraction AND one of the following: RVEDV/BSA ≥110 mL/m^2^ (male), ≥100 mL/m^2^ (female) OR RVEF ≤40% *Major (by RV angiography)* Regional RV akinesia, dyskinesia, or dyssynchronous RV contraction *Minor (by 2D echo)* Regional RV akinesia OR dyskinesia AND one of the following (end diastole): PLAX RVOT ≥29–≤32 mm (PLAX/BSA ≥16–≤19 mm/m^2^) PSAX RVOT ≥32–≤36 mm (PSAX/BSA ≥18–≤21 mm/m^2^) OR FAC >33%–≤40% *Minor (by MRI)* Regional RV akinesia OR dyskinesia OR dyssynchronous RV contraction AND one of the following: RVEDV/BSA ≥100–<110 mL/m^2^ (male), ≥90–<100 mL/m^2^ (female) OR RVEF >40%–≤45%
Structural alterations	*Revised Task Force Criteria for the diagnosis of ARVC (Marcus* et al.^7^*):* *Major* Residual myocytes <60% by morphometric analysis, (or < 50% if estimated), with fibrous replacement of the RV free wall myocardium in at least 1 sample, with or without fatty replacement of tissue on EBM. *Minor* Residual myocytes 60–75% by morphometric analysis, (or 50% to 65% if estimated), with fibrous replacement of the RV free wall myocardium in at least 1 sample, with or without fatty replacement of tissue on EBM.	*Padua criteria (Corrado et al.* ^8^ *):* *Major* Transmural LGE (stria pattern) of ≥1 RV region(s) (inlet, outlet, and apex in 2 orthogonal views) *(by CE-CMR)* Fibrous replacement of the myocardium in ≥1 sample, with or without fatty tissue *(by EMB)*	*Major* Residual myocytes <60% by morphometric analysis, (or < 50% if estimated), with fibrous replacement of the RV free wall myocardium in at least 1 sample, with or without fatty replacement of tissue on EBM *Minor* Residual myocytes 60–75% by morphometric analysis, (or 50% to 65% if estimated), with fibrous replacement of the RV free wall myocardium in at least 1 sample, with or without fatty replacement of tissue on EBM	*Major* Fibrous replacement of the myocardium in ≥1 sample, with or without fatty tissue, at histology *Minor* Unequivocal RV LGE (confirmed in 2 orthogonal views) in ≥1 RV region(s) (excluding tricuspid valve)	*Major* Residual myocytes <60% by morphometric analysis, (or < 50% if estimated), with fibrous replacement of the RV free wall myocardium in at least 1 sample, with or without fatty replacement of tissue on EBM *Minor* Residual myocytes 60–75% by morphometric analysis, (or 50% to 65% if estimated), with fibrous replacement of the RV free wall myocardium in at least 1 sample, with or without fatty replacement of tissue on EBM
Repolarization abnormalities	*Revised Task Force Criteria for the diagnosis of ARVC (Marcus et al.* ^7^ *):* *Major* Inverted T waves in right precordial leads (V1, V2 and V3) or beyond in individuals > 14 years of age (in the absence of complete RBBB QRS ≥ 120 msecs) *Minor* Inverted T waves in leads V1 and V2 in individuals > 14 years of age (in the absence of complete RBBB), or in V4, V5, or V6 Inverted T waves in leads V1, V2, V3 and V4 in individuals > 14 years of age in the presence of complete RBBB	*Padua criteria (Corrado et al.* ^8^ *):* *Major* Inverted T waves in right precordial leads (V1, V2 and V3) or beyond in individuals with complete pubertal development (in the absence of complete RBBB) *Minor* Inverted T waves in leads V1 and V2 in individuals with completed pubertal development (in the absence of complete RBBB) Inverted T waves in V1, V2, V3 and V4 in individuals with completed pubertal development in the presence of complete RBBB	*Major* Inverted T waves in right precordial leads (V1, V2 and V3) or beyond in individuals > 14 years of age (in the absence of complete RBBB QRS ≥ 120 msecs) *Minor* Inverted T waves in leads V1 and V2 in individuals > 14 years of age (in the absence of complete RBBB), or in V4, V5, or V6 Inverted T waves in leads V1, V2, V3 and V4 in individuals > 14 years of age in the presence of complete RBBB	*Major* Negative T waves in right precordial leads (V1, V2, and V3) or beyond in individuals ≥14-year-old (in the absence of complete RBBB and not preceded by J-point/ST-segment elevation) *Minor* Negative T waves in leads V1 and V2 in males ≥14-year-old (in the absence of RBBB and not preceded by J-point/ST-segment elevation) Negative T waves beyond V3 in the presence of complete RBBB Negative T waves beyond V3 in individuals <14-year-old	*Major* Inverted T waves in right precordial leads (V1, V2 and V3) or beyond in individuals > 14 years of age (in the absence of complete RBBB QRS ≥ 120 msecs) *Minor* Inverted T waves in leads V1 and V2 in individuals > 14 years of age (in the absence of complete RBBB), or in V4, V5, or V6 Inverted T waves in leads V1, V2, V3 and V4 in individuals > 14 years of age in the presence of complete RBBB
Depolarization and conduction abnormalities	*Revised Task Force Criteria for the diagnosis of ARVC (Marcus et al.* ^7^ *):* *Major* Epsilon wave (reproducible low amplitude signals between end of QRS complex to onset of the T wave) in the right precordial leads (V1 to V3) *Minor* Late potentials by signal averaged ECG in at least one of three parameters in the absence of a QRS duration of ≥110 msecs on the standard ECG Filtered QRS duration (fQRS) ≥114 ms Duration of terminal QRS < 40 μV (LAS) ≥38 ms Root-mean-square voltage of terminal 40 msecs ≥20 μV Terminal activation duration of QRS ≥ 55 ms measured from the nadir of the S wave to the end of the QRS, including R’, in V1, V2 or V3, in the absence of complete RBBB	*Padua criteria (Corrado et al.* ^8^ *):* *Minor* Epsilon wave (reproducible low amplitude signals between end of QRS complex to onset of the T wave) in the right precordial leads (V1 to V3) Terminal activation duration of QRS ≥ 55 ms measured from the nadir of the S wave to the end of the QRS, including R’, in V1, V2 or V3 (in the absence of complete RBBB)	*Major* Epsilon wave (reproducible low amplitude signals between end of QRS complex to onset of the T wave) in the right precordial leads (V1 to V3) *Minor* Late potentials by signal averaged ECG in at least one of three parameters in the absence of a QRS duration of ≥110 msecs on the standard ECG. Filtered QRS duration (fQRS) ≥114 ms Duration of terminal QRS < 40 μV (LAS) ≥38 ms Root-mean-square voltage of terminal 40 msecs ≥20 μV Terminal activation duration of QRS ≥ 55 ms measured from the nadir of the S wave to the end of the QRS, including R’, in V1, V2 or V3, in the absence of complete RBBB	*Minor* Epsilon wave (reproducible low-amplitude signals between end of QRS complex to onset of the T wave) in the right precordial leads (V1 to V3) Terminal activation duration of QRS ≥55 ms measured from the nadir of the S wave to the end of the QRS, including R’, in V1, V2, or V3 (in the absence of complete RBBB)	*Major* Epsilon wave (reproducible low amplitude signals between end of QRS complex to onset of the T wave) in the right precordial leads (V1 to V3) *Minor* Late potentials by signal averaged ECG in at least one of three parameters in the absence of a QRS duration of ≥110 msecs on the standard ECG: Filtered QRS duration (fQRS) ≥114 ms Duration of terminal QRS < 40 μV (LAS) ≥38 ms Root-mean-square voltage of terminal 40 msecs ≥20 μV Terminal activation duration of QRS ≥ 55 ms measured from the nadir of the S wave to the end of the QRS, including R’, in V1, V2 or V3, in the absence of complete RBBB
Arrhythmias	*Revised Task Force Criteria for the diagnosis of ARVC (Marcus et al.* ^7^ *):* *Major* Non-sustained or sustained VT of left bundle branch morphology with superior axis (negative or indeterminate QRS in II, III, AVF and positive in AVL) *Minor* Non-sustained or sustained VT of RVOT configuration, LBBB morphology with inferior axis (positive QRS in II, III, AVF and negative in AVL) or of unknown axis Greater than 500 ventricular extrasystoles/24 h by Holter	*Padua criteria (Corrado et al.* ^8^ *):* *Major* Frequent VEs (>500 per 24 h) or non-sustained or sustained VT of LBBB morphology *Minor* Frequent VEs (>500 per 24 h) or non-sustained or sustained VT of LBBB morphology with inferior axis (‘RVOT pattern’)	*Major* Non-sustained or sustained VT of left bundle branch morphology with superior axis (negative or indeterminate QRS in II, III, AVF and positive in AVL) *Minor* Non-sustained or sustained VT of RVOT configuration, LBBB morphology with inferior axis (positive QRS in II, III, AVF and negative in AVL) or of unknown axis Greater than 500 VEss/24 h by Holter	*Major* Frequent VEs (>500 per 24 h), non-sustained or sustained VT of LBBB morphology with non-inferior axis *Minor* Frequent VEs (>500 per 24 h), non-sustained or sustained VT of LBBB morphology with inferior axis (‘RVOT pattern’) History of cardiac arrest due to VF or sustained VT of unknown morphology	*Major* Non-sustained or sustained VT of left bundle branch morphology with superior axis (negative or indeterminate QRS in II, III, AVF and positive in AVL) *Minor* Non-sustained or sustained VT of RVOT configuration, LBBB morphology with inferior axis (positive QRS in II, III, AVF and negative in AVL) or of unknown axis Greater than 500 VEs/24 h by Holter
Family history/genetics	*Revised Task Force Criteria for the diagnosis of ARVC (Marcus et al.* ^7^ *):* *Major* ARVC confirmed in a first-degree relative who meets current task force criteria ARVC confirmed pathologically at autopsy or surgery in a first degree relative Identification of a pathogenic mutation categorized as associated or probably associated with ARVC in the patient under evaluation *Minor* History of ARVC in a first degree relative in whom it is not possible or practical to determine if the family member meets diagnostic criteria Premature sudden death (<35 years) due to suspected ARVC in a first degree relative ARVC confirmed pathologically or by diagnostic criteria in second degree	*Padua criteria (Corrado et al.* ^8^ *):* *Major* ACM confirmed in a first-degree relative who meets diagnostic criteria ACM confirmed pathologically at autopsy or surgery in a first-degree relative Identification of a pathogenic or likely pathogenetic ACM mutation in the patient under evaluation *Minor* History of ACM in a first-degree relative in whom it is not possible or practical to determine whether the family member meets diagnostic criteria Premature sudden death (<35 years) due to suspected ACM in a first degree relative ACM confirmed pathologically or by diagnostic criteria in second-degree relative	*Major* ARVC confirmed in a first-degree relative who meets diagnostic criteria ARVC confirmed pathologically at autopsy or surgery in a first-degree relative Identification of a pathogenic mutation categorized as associated or probably associated with ARVC in the patient under evaluation *Minor* History of ARVC in a first degree relative in whom it is not possible or practical to determine if the family member meets diagnostic criteria Premature sudden death (<35 years) due to suspected ARVC/D in a first degree relative ARVC/D confirmed pathologically or by diagnostic criteria in second-degree relative	*Major* Identification of a pathogenic ACM-gene variant in the patient under evaluation ACM confirmed in a first-degree relative who meets diagnostic criteria ACM confirmed pathologically at autopsy or surgery in a first-degree relative *Minor* Identification of a likely-pathogenic ACM-gene variant in the patient under evaluation History of ACM in a first-degree relative in whom it is not possible or practical to determine whether the family member meets diagnostic criteria Premature sudden death (<35 years of age) due to suspected ACM in a first-degree relative ACM confirmed pathologically or by diagnostic criteria in second-degree relative	*Major* ARVC confirmed in a first-degree relative who meets diagnostic criteria ARVC confirmed pathologically at autopsy or surgery in a first-degree relative Identification of a pathogenic mutation categorized as associated or probably associated with ARVC in the patient under evaluation *Minor* History of ARVC in a first-degree relative in whom it is not possible or practical to determine if the family member meets diagnostic criteria Premature sudden death (<35 years) due to suspected ARVC in a first-degree relative ARVC confirmed pathologically or by current Task Force Criteria in second-degree relative
Diagnostic criteria for ALVC	The NDLVC phenotype include ALVC, left-dominant ARVC, HNDC, or arrhythmogenic DCM The term NDLVC defined by the presence of non-ischaemic LV scarring or fatty replacement regardless of the presence of global or regional wall motion abnormalities, or isolated global LV hypokinesia without scarring. General endorsement of Padua criteria (Corrado *et al.*^8^), with acknowledgment of lack of external validation: No major or minor morpho-functional and/or structural RV criteria + ≥1 major structural LV criteria + Pathogenic or likely pathogenic ACM-causing gene mutation		NR	NR	European Task Force Proposed Diagnostic Criteria for the diagnosis of ACM (Corrado *et al.* ^1^ ): No major or minor morpho-functional or structural (tissue-characterisation) RV criteria + *Definite*: 2 major OR 1 major + 2 minor OR 4 minor criteria for ALVC from different categories (at least 1major or minor structural LV criteria) *Borderline*: 1 major + 1 minor OR 3 minor criteria for ALVC from different categories (at least 1 major or minor structural LV criteria) *Possible*: 1 major OR 2 minor criteria for ALVC from different categories (at least 1 major or minor structural LV criteria)
Morpho-functional ventricular abnormalities	*Major (by 2D echo, CMR or angiography)* Global LV systolic dysfunction (depression of LVEF or reduction of echocardiographic global longitudinal strain), with or without LV dilatation (increase of LVEDV according to the imaging test specific nomograms for age, sex, and BSA) *Minor (by 2D echo, CMR or angiography)* Regional LV hypokinesia or akinesia of LV free wall, septum, or both		NR	NR	*Minor (by 2D echo, CMR or angiography)* Global LV systolic dysfunction, with or without LV dilatation (increase of LVEDV according to the imaging test specific nomograms for age, sex, and BSA; .e. CMR: EDV/BSA >96 mL/m^2^ for women or >105 mL/m^2^ for men or >122 mL/m^2^ for athletes; LV EF <57% for women and men or <58% for athletes)
Structural alterations	*Major (by CE-CMR)* LV LGE (stria pattern) of ≥1 Bull's Eye segment(s) (in 2 orthogonal views) of the free wall (subepicardial or midmyocardial), septum, or both (excluding septal junctional LGE)		**NR**	Diagnostic criteria for LV involvement: not defined. If present, LGE is typically found in a subepicardial, or mid-wall distribution confined to the LV.	*Major (by CE-CMR)* ‘Ring-like’ LV LGE (subepicardial or midmyocardial stria pattern) of ≥3 segments (confirmed in 2 orthogonal views) *Minor (by CE-CMR)* LV LGE (subepicardial or midmyocardial stria pattern) of 1 or 2 Bull’s Eye segment(s) (in 2 orthogonal views) of the free wall, septum, or both (excluding patchy, focal or septal junctional LGE)
Repolarization abnormalities	*Minor* Inverted T waves in left precordial leads (V4-V6) (in the absence of complete LBBB)		NR	NR	*Minor* Negative T waves in left precordial leads (V4-V6) (in the absence of complete LBBB)
Depolarization and Conduction abnormalities	*Minor* Low QRS voltages (<0.5 mV peak to peak) in limb leads (in the absence of obesity, emphysema, or pericardial effusion)		NR	NR	*Major* Low QRS voltages (<0.5 mV peak to peak) in all limb leads in the absence of other causes (e.g. cardiac amyloidosis, obesity, emphysema, or pericardial effusion)
Arrhythmias	*Minor* Frequent VEs (>500 per 24 h), non-sustained or sustained VT with a RBBB morphology (excluding the ‘fascicular pattern’)		NR	NR	*Minor* Frequent (>500 per 24 h) or exercise-induced VEs with a RBBB morphology or multiple RBBB morphologies (excluding the ‘fascicular pattern’) Non-sustained or sustained VT with a RBBB morphology (excluding the ‘fascicular pattern’) History of cardiac arrest due to VF or sustained VT of unknown morphology
Family history/genetics	*Major* ACM confirmed in a first-degree relative who meets diagnostic criteria ACM confirmed pathologically at autopsy or surgery in a first-degree relative Identification of a pathogenic or likely pathogenetic ACM mutation in the patient under evaluation *Minor* History of ACM in a first-degree relative in whom it is not possible or practical to determine whether the family member meets diagnostic criteria Premature sudden death (<35 years) due to suspected ACM in a first degree relative ACM confirmed pathologically or by diagnostic criteria in second-degree relative		NR	NR	*Major* Identification of a pathogenic ACM-gene variant in the patient under evaluation ACM confirmed in a first-degree relative who meets diagnostic criteria ACM confirmed pathologically at autopsy or surgery in a first-degree relative *Minor* Identification of a likely-pathogenic ACM-gene variant in the patient under evaluation History of ACM in a first-degree relative in whom it is not possible or practical to determine whether the family member meets diagnostic criteria Premature sudden death (<35 years of age) due to suspected ACM in a first-degree relative ACM confirmed pathologically or by diagnostic criteria in second-degree relative
Diagnostic criteria for biventricular ACM	General endorsement of Padua criteria (Corrado *et al.* ^8^ ), with acknowledgment of lack of external validation: ≥1 major or minor morpho-functional and/or structural RV criteria + ≥1 major or minor morpho-functional or structural (tissue-characterisation) LV criteria + *Definite*: 2 major OR 1 major + 2 minor OR 4 minor RV and LV criteria from different categories *Borderline*: 1 major + 2 minor OR 3 minor RV and LV criteria from different categories *Possible*: 1 major OR 2 minor RV and LV criteria from different categories		NR	NR	European Task Force Proposed Diagnostic Criteria for the diagnosis of ACM (Corrado *et al.* ^1^ ): ≥1 major or minor morpho-functional or structural (tissue-characterisation) RV criteria + ≥1 major or minor morpho-functional or structural (tissue-characterisation) LV criteria *Definite*: 2 major OR 1 major + 2 minor OR 4 minor criteria for ARVC and ALVC from different categories *Borderline*: 1 major + 1 minor OR 3 minor criteria for ARVC and ALVC from different categories *Possible*: 1 major OR 2 minor criteria for ARVC and ALVC from different categories

ACC, American College of Cardiology; ACM, arrhythmogenic cardiomyopathy; AHA, American Heart Association; ALVC, arrhythmogenic left ventricular cardiomyopathy; ARVC, arrhythmogenic right ventricular cardiomyopathy; BSA, body surface area; CE-CMR, contrast-enhancement cardiac magnetic resonance; CMR, cardiac magnetic resonance; DCM, dilated cardiomyopathy; EBM, endomyocardial biopsy; ECG, electrocardiogram; EDV, end-diastolic volume; ESC, European Society of Cardiology; ETF/ITF, European/International Task Force; FAC, fractional area change; HRS, Heart Rhythm Society; LAS, low amplitude signal; LBBB, left bundle branch block; LGE, late gadolinium enhancement; LV, left ventricle; LVEDV, left ventricular end-diastolic volume; LVEF, left ventricle ejection fraction; MRI, magnetic resonance imaging; NR, not reported; PLAX, parasternal long axis; PSAX, parasternal short axis; RBBB, right bundle branch block; RV, right ventricle; RVEDV, right ventricular end-diastolic volume; RVEF, right ventricle ejection fraction; RVOT, right ventricular outflow tract; SCA, sudden cardiac arrest; TWI, T wave inversion; VE, ventricular extrasystole; VF, ventricular fibrillation; VT, ventricular tachycardia.

**Table 3:** Diagnostic criteria for arrhythmogenic cardiomyopathy and their recommendation according to different societies

Diagnostic criteria	Organization society
Revised Task Force Criteria for the diagnosis of ARVC (Marcus *et al.*^7^)	ESC^9^, AHA/ACC/HRS^11^, HRS^12^
Padua criteria (Corrado *et al.*^8^)	ESC^9^ provides general endorsement for the Padua criteria but acknowledges the lack of external validation
European Task Force Proposed Diagnostic Criteria for the diagnosis of ACM (Corrado *et al.*^1^)	ETF/ITF^1^

ACC, American College of Cardiology; AHA, American Heart Association; ALVC, arrhythmogenic left ventricular cardiomyopathy; ARVC, arrhythmogenic right ventricular cardiomyopathy; ESC, European Society of Cardiology; ETF/ITF, European/International Task Force; HRS, Heart Rhythm Society.

#### Phenocopies

There is disagreement among the different guidelines and recommendations regarding the definition of ACM phenocopies. Specifically, the AHA/ACC does not address this issue, while the ESC recognizes phenocopies in both structural and non-structural diseases.^9^ The ETF/ITF takes this further by distinguishing between ARVC phenocopies, ALVC phenocopies, and BivACM phenocopies.^1^ In contrast, the HRS does not categorize these conditions as phenocopies but instead considers certain systemic disorders (e.g. sarcoidosis, amyloidosis), myocarditis, and Chagas disease as important causes of ACM.^12^

#### Genetic counselling/testing, screening, follow-up and management of family members

While there is consensus among the different guidelines regarding genetic management for index patients, inconsistencies arise concerning the management of family members. First-degree relatives of patients with ARVC and a disease-causing variant are indicated for genetic testing with a Class I recommendation by the ESC, AHA/ACC, and HRS.^9,11,12^ If the relatives test negative for the familial variant, they may be discharged from follow-up, whereas those who test positive are enrolled in long-term follow-up. The ESC additionally provides a Class IIa indication for genetic testing in affected first-degree relatives of an ARVC patient with a variant of uncertain significance (VUS), for segregation purposes,^9^ a recommendation not addressed by other societies. Moreover, the ESC advises long-term follow-up (Class I) or repeated clinical and imaging screening (Class IIa) for first-degree relatives of an ARVC patient who has not undergone genetic testing but presents with either a cardiomyopathy phenotype or a normal phenotype, respectively.^9^ Other societies do not offer guidance on these aspects of family cascade screening.

Regarding the divergence in exercise recommendations for family members, only genotype-positive/phenotype-negative individuals from ARVC families are addressed against high-intensity exercise: specifically, the ESC advises against such activity with a Class IIb indication,^9^ while the ETF/ITF recommends avoidance with a Class IIa indication.^10^ Other societies do not address this issue.

#### Management of ventricular arrhythmias: other anti-arrhythmic drugs

The management of VAs is consistently outlined across various guidelines, with an emphasis on the strategic use of beta-blockers and transcatheter ablation, favouring endo-epicardial approaches performed in specialized centres. While beta-blockers remain the cornerstone of therapy, the recommendations for other antiarrhythmic agents are less uniform. The ESC grants amiodarone a Class IIa indication for patients who do not respond adequately to beta-blockers,^9^ whereas the HRS provides a Class IIb indication, suggesting its use in individuals with persistent arrhythmic symptoms or to decrease interventions by ICDs.^12^ Flecainide is similarly endorsed with a Class IIa recommendation by the ESC,^9^ while the HRS assigns it a Class IIb status, advocating its use alongside beta-blockers when monotherapy proves insufficient.^12^

#### ICD implantation for primary prevention

For primary prevention, ICD implantation is recommended, irrespective of the presence of VAs, in patients with severe right ventricular dysfunction and a LVEF ≤ 35%, with general consensus across all guidelines.^9–12^ Additionally, the ETF/ITF and AHA/ACC provide a Class I recommendation for ICD implantation in primary prevention when the right ventricular ejection fraction is ≤35%.^10,11^ The ETF/ITF classifies ARVC patients into high, intermediate, and low-risk categories based on the estimated annual incidence of life-threatening arrhythmic events (≥10%, 1–10%, and <1%, respectively). This classification depends on factors such as the presence of VAs, ventricular systolic dysfunction, history of syncope, sex, complex genotypes, and endocardial voltage mapping. Implantable cardiac defibrillator implantation is recommended with a Class I indication for high-risk patients, Class IIa or IIb for intermediate-risk individuals depending on risk factors, and contraindicated (Class III) for low-risk patients.^10^

Both the HRS and ESC, when considering NDLVC encompassing ALVC and BivACM, recommend ICD implantation for ACM patients with LVEF > 35% who carry high-risk genotypes. The ESC further identifies six specific genotypes (*LMNA, FLNC*-truncating variants, *TMEM43, PLN, DSP* and *RBM20*). However, the two documents differ in their class of recommendation and specific risk factors for SCD. These include LVEF <45%, sex, and non-sustained VT (NSVT), with the ESC also considering LGE on CMR and gene-specific risk scores for *LMNA* and p.Arg14del variants of *PLN*.^9,12^ Notably, only the ESC includes the updated 2019 ARVC risk calculator to assist with shared decision-making, especially in gene-positive ARVC and particularly for *PKP2*.^9^ Both the ESC and ETF/ITF provide guidance on selecting the appropriate type of device for ICD implantation: the ESC^9^ recommends subcutaneous ICD when pacing therapy for bradycardia, cardiac resynchronization therapy or anti-tachycardia pacing is not anticipated(IIa), while ETF/ITF^10^ suggests considering either a single-chamber ICD or a subcutaneous ICD. Currently, no specific recommendations exist for variants at high risk of conduction system disease, such as laminopathy, where transvenous devices should be preferred.

#### Guidance for comprehensive care

Only the ESC guidelines provide specific recommendations for access to multidisciplinary team, with emphasis on psychological support for patients and family members, the management of atrial fibrillation and flutter, non-cardiac surgery, and reproductive issues.^9^

## Discussion

We identified 5 rigorously developed documents that provide comprehensive guidance on the diagnosis and management of ACM. These include two guidelines^9,11^ and three consensus statements.^1,10,12^ While clinical statements may rely more on consensus than robust evidence and often do not undergo the same development process as formal guidelines, they still reflect the collective expertise of leading clinicians. In the specific case of ACM, where randomized trials are lacking, even the recommendations outlined in formal guidelines are largely derived from observational studies, single trials or non-randomized studies, follow-up registries, and expert opinions and fall under Class B or C evidence. The inclusion of consensus statements may in the short-term add additional insights in the absence of adequate evidence-based recommendations.

The documents consistently emphasize the critical role of echocardiography and CMR in diagnosing, monitoring, and risk stratifying patients with ACM, in alignment with all proposed diagnostic criteria. Furthermore, there is consensus regarding genetic testing for ACM patients, ideally coupled with genetic counselling provided by specialists in both genetics and cardiology with extensive expertise in clinical interpretation of genetic variants.

Despite the identification of several specific genetic causes, the significant variability in disease penetrance underscores the critical role of environmental factors, especially high-intensity physical activity, in the pathogenesis of ACM.^17,18^ Physical exercise seems to further stress and worsen the cell-to-cell adhesion defect, particularly affecting the thinner-walled right ventricle more than the left ventricle.^3^ Consequently, there is consensus across all societies that patients with ACM should abstain from high-intensity physical activities and competitive sports to mitigate the risk of disease progression and prevent complications.

However, discrepancies were identified in several areas, including the definition of ACM, the selection of diagnostic criteria, the interpretation of phenocopies, the management of family members, use of antiarrhythmic agents, indications for ICD implantation, and specific recommendations related to multidisciplinary teaming, atrial fibrillation, non-cardiac surgery and reproductive issues.

Key discrepancies in the definition of ACM across the reviewed documents pertain mainly to its left-dominant and biventricular forms, reflecting the evolving understanding of ACM over recent decades. Originally characterized as ‘arrhythmogenic right ventricular dysplasia’ (ARVD) by Marcus and Thiene, the condition was identified through the association of sudden cardiac death (SCD) during exertion in young adults with right ventricular (RV) abnormalities. These abnormalities were typified by progressive myocardial loss in the RV, coupled with inflammation, degeneration, and necrosis.^2,19^ The subsequent identification of mutations in genes coding for desmosomal proteins prompted a terminological transition from ‘dysplasia’ to ‘cardiomyopathy,’ acknowledging the genetic underpinnings of the disease.^20^ With advances in CMR imaging and genetic research, LV involvement in ACM was increasingly recognized, culminating in the publication of the Padua Criteria in 2020, which formally acknowledged a more inclusive, biventricular perspective, integrating both structural and molecular insights to refine the diagnostic framework.^8^

The AHA/ACC and HRS issued their documents^11,12^ prior to the publication of the Padua Criteria,^8^ and therefore focus primarily on ARVC, while acknowledging potential LV involvement. Although the ESC published their guidelines^9^ after the Padua Criteria,^8^ they offer specific recommendations for ARVC and address left-dominant and biventricular forms within the NDLVC section.^9^

The Padua criteria^8^ introduced several significant innovations in the diagnosis of ACM. These criteria established specific diagnostic benchmarks for ALVC and BivACM, acknowledging the diverse phenotypic spectrum of the disease. A notable inclusion was the use of LGE as a structural marker, which has expanded the role of imaging diagnostics and consequently reduced reliance on endomyocardial biopsy (EMB) in ACM diagnosis. EMB has a significant false-negative rate due to the patchy and uneven distribution of fibrofatty replacement, which increases the likelihood of sampling unaffected tissue. Additionally, biopsy of the right ventricular free wall is associated with a high risk of perforation due to its thin structure. However, both the ESC guidelines^9^ and the HRS consensus statement^12^ recommend EMB for differentiating myocardial inflammation (e.g. sarcoidosis), recognizing its limitations in detecting patchy involvement and highlighting the importance of electroanatomical voltage mapping-guided EMB.^21,22^ The criteria also adopted a two-step approach: initially assessing the presence of major and minor indicators for RV and LV involvement, followed by categorizing the ACM phenotype into three distinct variants—classic right-dominant ARVC, biventricular ACM, or left-dominant ALVC—based on these findings. Another innovation was the adoption of reference values for ventricular size and systolic function, tailored according to nomograms that adjust for age, sex, and BSA, moving away from outdated, fixed thresholds based on gradient echo cine sequences.^7^ Additionally, the epsilon wave was reclassified from a major to a minor ECG criterion, reflecting its variable diagnostic sensitivity. Further, the Padua criteria acknowledged post-mortem diagnosis of ACM in a first-degree relative as a major genetic indicator, highlighting the importance of family history.

The 2024 ETF criteria represent a further refinement of Padua criteria.^1^ Key differences include the reclassification of LGE from a major to a minor structural criterion, except for ‘ring-like’ LV LGE, which is still considered as major. Similarly, the classification of likely-pathogenic ACM variants has been downgraded from a major to a minor genetic criterion. Perhaps most notably, the ETF criteria have removed the mandatory requirement for ACM-gene mutations in the diagnosis of ALVC, allowing for a broader, phenotype-based diagnostic approach. Both the 2020 Padua criteria^8^ and the 2024 ETF criteria^1^ lack structural criteria derived from EMB for assessing left ventricular involvement in ACM.

**Figure 2:**
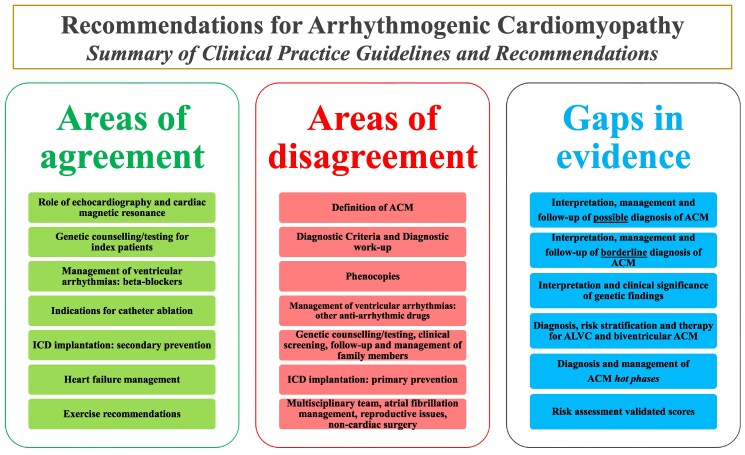
Summary of clinical practice guidelines and recommendations on arrhythmogenic cardiomyopathy diagnosis and management. ACM, arrhythmogenic cardiomyopathy; ALVC, arrhythmogenic left ventricular cardiomyopathy; ICD, implantable cardiac defibrillator.

*Figure 2* provides a summary of the areas of agreement, disagreement, and evidence gaps that may inform future research and guideline development.

### Gaps in ACM management

Despite the advancements in understanding ACM, several key areas remain inadequately addressed. One major gap is the lack of clinical guidelines for managing possible and borderline ACM diagnoses, as current documents are primarily focused on patients with a definitive diagnosis. Furthermore, the role of genetics in ACM etiopathogenesis remains unclear, although associations between pathogenic or likely pathogenic variants and ACM phenotypes have been observed. There is also an unmet need for specific guidance on managing ALVC and BivACM, both of which are recognized in the Padua^8^ and ETF criteria,^1^ but lack recommendations on complication prevention and management.

Moreover, the reviewed documents fail to address the ‘hot phases’ of ACM, an uncommon but clinically relevant presentation characterized by myocarditis-like syndrome, including chest pain, ECG changes, and elevated markers of myocardial necrosis.^23^ ‘Hot-phases’ may be the first manifestation of the disease and may have important prognostic implications. Currently, the role of ‘hot-phase’ episodes in disease progression and arrhythmic risk stratification remains to be clarified. Arrhythmogenic cardiomyopathy also lacks validated risk assessment tools and scoring systems, except for the ESC guidelines, which incorporate the updated 2019 ARVC risk calculator and *PLN/LMNA* risk scores to assist in ICD implantation decisions for primary prevention.^9^ Furthermore, aetiology-specific treatments remain a major gap, as most available therapies do not directly target the underlying causes of the disease.

#### Possible and borderline diagnosis of ACM

All the reviewed diagnostic criteria, despite their variations, consistently classify ACM diagnosis into definite, possible, or borderline, depending on the number of major or minor criteria present.^1,7,8^ However, there is a notable absence of guidance on how to manage patients with possible or borderline ACM. The literature emphasizes the importance of regular follow-up and reassessment in these cases to detect the development of new criteria that could confirm a definite diagnosis.^1^ Nevertheless, there are no clear recommendations on the frequency of follow-up or the tests that should be repeated. Additionally, there is no consensus on whether or how to screen family members of patients with possible or borderline ACM, despite the potential for some relatives to already meet the criteria for a definitive diagnosis.

#### Clinical significance of genetic findings

All proposed diagnostic criteria for ACM include genetic findings, categorized as major or minor depending on the type of mutation detected.^1,7,8^ However, the pathogenicity of many genetic variants is still poorly understood, requiring caution to prevent misdiagnosis. Many variants considered disease-associated are also found in the general population, contributing to the high ‘genetic noise’.^24^

ACM exhibits significant genetic heterogeneity, with mutations classified as pathogenic, likely pathogenic, or VUS. Various genotypes have been linked to right, left, or biventricular ACM phenotypes.^1^ Early genetic discoveries in ACM included syndromic homozygous forms like Naxos disease and Carvajal syndrome, linked to desmosomal protein mutations in *JUP* and *DSP*.^25,26^ Further research identified additional genes involved in non-syndromic ACM, including those encoding desmosomal proteins (*PKP2,DSP,DSG2,DSC2,JUP*), adherens junction proteins (*CTNNA3,CDH2*), components of the nuclear envelope (*LMNA,TMEM43*), intermediate filaments (*DES*), and transmembrane proteins (*PLN,SCN5A*).^3^ The variability in penetrance and the influence of environmental factors suggest that epigenetics may also play a role, although experimental evidence remains limited. Although most variants are inherited in an autosomal dominant pattern, there are also recessive patterns, as well as dominant patterns involving compound heterozygosity, digenic inheritance and varying degrees of penetrance influenced by age, gender, and physical activity. Alongside mutations already known to be associated with the ACM phenotype, there are numerous cases of ACM with clear familial transmission in which, however, a causal gene cannot be identified, so-called ‘familial but gene-elusive’ conditions.^3^ Furthermore, several mutations have been found in various genes without a clear genotype-phenotype correlation, often classified as VUS.^27^ Both gene-elusive forms and VUS present a significant challenge in both diagnosis and management, as the absence of a clear genetic marker complicates genetic counselling and risk assessment for family members. Despite their clinical importance, these gene-elusive forms and VUS are not addressed in the current guidelines, underscoring the need for more comprehensive recommendations. A deeper understanding of the genetic mechanisms underlying ACM could pave the way for the development of targeted therapies aimed at correcting the genetic defect, potentially preventing its progression or curing the disease. Research on potential therapies based on adenovirus-mediated gene delivery and CRISPR-Cas9 technology in animal models is underway.^28,29^

#### Left dominant and biventricular ACM

Although left or biventricular involvement in ACM is acknowledged by all major societies,^1,10–12^ most therapeutic guidelines continue to focus primarily on ARVC. The ESC takes a phenotypic approach, identifying two main ACM-related phenotypes: ARVC and NDLVC, with the latter encompassing ALVC, left-dominant ARVC, and BivACM.^9^ NDLVC is characterized by non-ischaemic LV scarring or fatty replacement, irrespective of the presence of global or regional wall motion abnormalities, or isolated global LV hypokinesia without scarring. While LV dilation and systolic dysfunction are typically absent, the ESC does not provide explicit cut-offs for these features. In contrast, the Padua criteria account for LV dilation in ALVC and BivACM cases, which often tracks disease progression.^30^ Dilated ACM phenotypes—though rarer than other ACM variants—are likely a result of disease progression, marked by increasing LGE affecting multiple segments of the LV free wall and septum, with more extensive involvement.^30^ These dilated phenotypes would be categorized by the ESC under dilated cardiomyopathy(DCM) rather than NDLVC, even though ACM often differs from DCM in terms of aetiology and pathogenesis, particularly in the relationship between the extent of myocardial fibrosis and degree of LV dysfunction.^31^ Moreover, phenotypic heterogeneity is frequently observed in overlapping genotypes, such as filamin C, a genotype typically associated with left ventricular expression in both dilated and arrhythmogenic forms. Thus, the distinction between dilated and non-dilated forms of ACM and among ACM, DCM and NDLVC highlights the complexity of categorizing these phenotypes and underscores the need for a more nuanced approach in clinical practice. Of note, the cardiomyopathy phenotype, according to the ESC, should not be regarded as a distinct diagnostic entity but rather as an entry point into a broader clinical diagnostic workflow aimed at phenotype-based integrated aetiological diagnosis. As an example, the ESC guidelines present the case of a 17-year-old male with palpitations, a family history of sudden cardiac death, subepicardial LGE, and a desmoplakin mutation. While the ESC diagnose the patient with *DSP-related NDLVC with subepicardial scar and low-normal EF*, the ETF/ITF would categorize the same patient as having *definite ALVC* based on 1 major criterion (pathogenic *DSP* mutation) and 3 minor criteria (family history of sudden death, regional LGE of the left ventricle, and LV systolic dysfunction).^9^ At present, the categorization of the same patient into two different diagnostic groups, NDLVC by the ESC and ALVC by the ETF, may have little impact on clinical management. Indeed, the ESC recommendations for the broader NDLVC category primarily focus on indications for ICD implantation.^9^ In contrast, the ETF/ITF, while extensively acknowledging left and biventricular forms of ACM in their diagnostic criteria,^1^ still rely on the 2015 document for ARVC therapeutic recommendations, a time when these phenotypes had not yet been fully recognized.^10^ The AHA/ACC also does not provide specific recommendations for ALVC and BivACM, while the HRS makes only occasional references to these subtypes.^12^ The lack of comprehensive and evidence-based guidance for management of NDLVC, ALVC and BivACM highlights the necessity of a standardized and unified approach to provide consistent and effective treatment strategies for these ACM subtypes.

#### Diagnosis and management of ACM ‘hot phases’

ACM can occasionally present with a myocarditis-like syndrome, known as a ‘hot phase’, characterized by chest pain, ECG changes, and elevated myocardial injury markers. These episodes may represent the initial manifestation of ACM or occur in previously diagnosed patients. In ACM patients with hot phase episodes, *DSP* mutations are often identified.^23,32^ Although not explicitly recommended in current guidelines, recognizing this presentation is important for prognosis.

The underlying pathophysiology of hot phases remains unclear, though it is hypothesized that genetically determined desmosomal dysfunction triggers cardiomyocyte death, which is exacerbated by an inflammatory response. This ‘double-hit’ hypothesis could lead to accelerated fibrofatty replacement of myocardial tissue.^3^ Studying these inflammatory episodes could help identify patients at higher risk or with more rapidly progressing disease. Additionally, understanding this phase may lead to new therapies aimed at reducing inflammation and mitigating arrhythmic risk.

### Limitations

Several limitations warrant consideration due to their potential for introducing bias. Our systematic review was confined to scientific literature published in English. We tried to reduce the impact of this restriction on our findings by adhering to a rigorous systematic methodology (PRISMA guidelines). We acknowledge that scientific statements often rely more heavily on consensus than on rigorous evidence and may not undergo the same development process as formal guidelines, they nonetheless reflect collective expertise of clinicians with an interest in these areas, especially where formal guidelines may be lacking. Specific recommendations regarding the management of comorbidities associated with ACM were not evaluated, as this falls outside the scope of our review. Also, we did not evaluate the validity of individual recommendations presented in the guidelines and scientific documents. However, we ensured that all included documents adhered to a defined standard of rigour, as assessed using the AGREE II tool.

## Conclusions

There is concordance among clinical practice guidelines and consensus recommendations regarding the use of cardiac imaging, particularly echocardiography and CMR, in the diagnostic assessment and follow-up of patients with ACM. Similar agreement is found on the importance of genetic counselling and testing in index patients, the management of ventricular arrhythmias, indications for catheter ablation, heart failure management, and exercise recommendations. There is a notable variation on the definition of ACM, the diagnostic criteria to be applied, and the classification of phenocopies and genocopies. Moreover, variations exist in the management of family members and the indications for ICD implantation. Further research is crucial to better understand the pathogenesis of ACM, particularly its genetic mechanisms, which could serve as therapeutic targets in the future. Significant gaps remain and most recommendations regarding ACM are predominantly informed by registries and expert opinions, underscoring the critical need for randomized trials to establish robust, evidence-based guidelines. Finally, evidence-based recommendations are needed for managing ALVC and BivACM, which are currently underrepresented in international guidelines.

## Supplementary Material

qcaf029_Supplementary_Data

## Data Availability

The data underlying this article are available in the manuscript and in the online Supplementary material. Any other data can be made available on reasonable request to the corresponding author.
